# Regulation of B cell receptor signalling by Epstein–Barr virus nuclear antigens

**DOI:** 10.1042/BCJ20220417

**Published:** 2022-12-07

**Authors:** Sarika Khasnis, Hildegonda Veenstra, Michael J. McClellan, Opeoluwa Ojeniyi, C. David Wood, Michelle J. West

**Affiliations:** School of Life Sciences, University of Sussex, Brighton BN1 9QG, U.K.

**Keywords:** b cell receptor signalling, Epstein–Barr virus, signalling, transcription factors

## Abstract

The cancer-associated Epstein–Barr virus (EBV) latently infects and immortalises B lymphocytes. EBV latent membrane protein 2A and EBV-encoded microRNAs are known to manipulate B cell receptor signalling to control cell growth and survival and suppress lytic replication. Here, we show that the EBV transcription factors EBNA2, 3A, 3B and 3C bind to genomic sites around multiple B cell receptor (BCR) pathway genes, regulate their expression and affect BCR signalling. EBNA2 regulates the majority of BCR pathway genes associated with binding sites, where EBNA3 proteins regulate only 42% of targets predicted by binding. Both EBNA2 and 3 proteins predominantly repress BCR pathway gene expression and target some common genes. EBNA2 and at least one EBNA3 protein repress the central BCR components *CD79A* and *CD79B* and the downstream genes *BLNK*, *CD22*, *CD72*, *NFATC1*, *PIK3CG* and *RASGRP3*. Studying repression of *CD79B*, we show that EBNA2 decreases transcription by disrupting binding of Early B cell Factor-1 to the *CD79B* promoter. Consistent with repression of BCR signalling, we demonstrate that EBNA2 and EBNA3 proteins suppress the basal or active BCR signalling that culminates in NFAT activation. Additionally, we show that EBNA2, EBNA3A and EBNA3C expression can result in reductions in the active serine 473 phosphorylated form of Akt in certain cell contexts, consistent with transcriptional repression of the PI3K-Akt BCR signalling arm. Overall, we identify EBNA2, EBNA3A and EBNA3C-mediated transcription control of BCR signalling as an additional strategy through which EBV may control the growth and survival of infected B cells and maintain viral latency.

## Introduction

Epstein–Barr virus (EBV) is a human gamma herpesvirus carried by more than 90% of the world population that persists in B lymphocytes. Infection normally occurs in childhood and is mostly asymptomatic, but if delayed until adolescence or adulthood, primary infection can cause infectious mononucleosis. EBV is also associated with B cell cancers including Burkitt, Hodgkin, post-transplant, AIDS-associated and diffuse large B cell lymphomas. The virus can also infect epithelial cells and is associated with the epithelial malignancies nasopharyngeal carcinoma and gastric cancer. While the mechanisms through which EBV drives lymphomagenesis are not fully defined, the ability of EBV to immortalise human B cells *in vitro* turning them into permanently growing lymphoblastoid cell lines (LCLs) is a key determinant of its oncogenicity. Like all herpes viruses, EBV has two phases in its life cycle: a latent phase and an acute productive lytic phase. When EBV infects naïve B cells it expresses its full repertoire of viral latent genes including six EBV nuclear antigens (EBNA1, 2, 3A, 3B, 3C and -LP), three latent membrane proteins (LMP1, 2A and 2B) and several functional small encoded RNAs (EBERs) and microRNAs (miRNAs) [1]. These latent genes collaboratively reprogramme the host B lymphocyte to drive growth, inhibit apoptosis and exploit the B cell differentiation pathway to access the site of long-term viral persistence, the memory B cell.

Transcriptional reprogramming by the EBV latent antigens EBNA2, EBNA3A, EBNA3B and EBNA3C plays a critical role in B cell immortalisation. These EBNAs do not bind gene regulatory elements directly and rely on interactions with cell transcription factors e.g. RBP-J (CBF-1), EBF-1 and CBFß to control the transcription of viral and cellular genes [2–6]. EBNA2 contains an acidic activation domain, associates with multiple general transcription factors and co-activators and is the major viral transactivator in latency [7]. It activates transcription of all EBNA and LMP genes through promoter-proximal elements in a largely RBP-J-dependent manner [2,3,8]. EBNA2 also binds gene promoter and enhancer elements across the B cell genome, with most binding at enhancer sites [9,10]. Activation of numerous cellular genes (e.g. *MYC*, *RUNX3*, *miR-155HG*) by EBNA2 involves reorganisation of three-dimensional interactions between promoters and enhancers to increase promoter contacts [9,11–13]. There is extensive overlap in EBNA2 and RBP-J binding in the B cell genome, indicating that RBP-J is also a major DNA-binding adaptor for host cell transcriptional reprogramming [9]. This supports the requirement for RBP-J binding by EBNA2 for B cell immortalisation [14]. Additional cellular DNA-binding proteins have also been implicated in gene regulation by EBNA2, including EBF-1 and PU.1 [5,15]. EBNA2 can also reduce transcription of IgM [16] and many other cell genes [17], but the mechanism through which EBNA2 represses transcription is unknown. Conversely, although the EBNA3 family of proteins (EBNA3A, 3B and 3C) can activate and repress transcription and associate with co-activators and corepressors, their transcriptional repression mechanisms are most well studied [18]. Like EBNA2, EBNA3 proteins cannot bind DNA directly and associate with genes through cell DNA-binding proteins including RBP-J, PU.1, IRF4 and CBFβ [6,19–23]. EBNA3 proteins can negatively regulate EBNA2-mediated gene activation as they competitively bind to RBP-J [24,25], but also independently repress transcription through polycomb-mediated silencing mechanisms [26–29]. In the B cell genome, EBNA3 proteins bind predominantly to long-range enhancers and manipulate enhancer–promoter looping to activate or repress transcription [10,11,30].

The B cell receptor (BCR) controls growth and apoptosis in normal and malignant B cells and perturbation of BCR signalling has been implicated in the pathogenesis of numerous leukaemias and lymphomas [31]. The BCR comprises a surface immunoglobulin non-covalently bonded to a heterodimer composed of CD79A (Igα) and CD79B (Igβ) that mediates signal transduction [32,33]. BCR activation leads to receptor aggregation and phosphorylation of tyrosine residues in the immunoreceptor tyrosine-based activation motifs (ITAMs) within the cytoplasmic tails of CD79A and CD79B by the tyrosine kinase Lyn. This phosphorylation forms a signalosome comprising the BCR, the kinases Syk, Btk and Lyn, the adaptor proteins Grb2 and B cell linker kinase (BLNK) and signalling components such as guanine exchange factor proteins Vav [1–3], PLCγ2 and PI3K resulting in activation of the calcium, MAPK and NF-κB survival and proliferation pathways [32]. Activation of PLCγ leads to the release of calcium, which is bound by calmodulin and results in activation of the calmodulin-dependent protein phosphatase calcineurin which dephosphorylates the nuclear factor of activated T cells (NFAT) transcription factors. NFATs then translocate to the nucleus, bind to partner proteins and activate target genes. PI3K activation leads to phosphorylation of Akt and activation of the NF-κB pathway, culminating in the translocation of the NF-κB transcription factor to the nucleus and the activation of target gene transcription.

The switch from EBV latency into lytic cycle is triggered by engagement of the BCR. EBV LMPs and miRNAs are known to manipulate BCR signalling to promote the growth and survival of latently infected B cells and to prevent lytic cycle activation. LMP2A has been shown to antagonise or mimic/enhance BCR signalling in a context-specific manner. In LMP2A expressing cells with a functional BCR, BCR cross-linking fails to activate key pathway components resulting in no calcium flux and failed activation of the EBV lytic cycle [34]. Since the cytoplasmic amino terminal domain of LMP2A contains an ITAM that binds Lyn and Syk, it has been proposed that LMP2A sequesters these kinases and prevents their activation by the BCR [34]. LMP2A has also been shown to prevent the BCR from entering lipid rafts, thus blocking signalling and antigen transport [35]. However, studies in transgenic mice have shown that in the absence of a functional BCR, LMP2A can also substitute for some BCR signals to drive the proliferation and survival of B cells [36,37]. Studies on antigen exposed mice have also demonstrated that expression of LMP2A can augment antibody responses [38]. In addition to BCR signalling manipulation by LMP2A, the EBV oncogene LMP1 and the EBV-encoded miRNAs have been shown to down-regulate expression of many BCR pathway genes. LMP1 expression in germinal centre B cells leads to *CD79A*, *CD79B*, *CD19*, *CD20*, *CD22* and *BLNK* down-regulation [39] and the BHRF1–2 miRNAs attenuate BCR signalling and lytic cycle activation by reducing expression of BCR pathway components including *GRB2*, *SOS1*, *MALT1*, *RAC1* and *INPP5D* [40].

Increasing evidence from transcriptomics and ChIP-sequencing analysis from our own and other laboratories indicates that EBNA2 and EBNA3 proteins also target and alter the expression of multiple genes in the BCR signalling pathway, but this has never been fully investigated [9,17,29,41–47]. We set out to confirm the regulation of BCR pathway genes by these EBNAs, examine the mechanisms involved and determine the functional consequences. We show that EBNA2 represses *CD79B* expression by binding to the promoter and interfering with binding and transcriptional activation by EBF-1. We also demonstrate that EBNA2, in addition to EBNA3A and EBNA3C, represses genes in the PI3K-Akt pathway in certain cell contexts leading to decreased activation of Akt. Consistent with the suppression of signalling from the BCR, we show that EBNA2 and EBNA3 expression in EBV-negative Burkitt lymphoma cells reduces NFAT-activated transcription. Our data, therefore, provide evidence that additional EBV-encoded genes may contribute to the attenuation of BCR signalling in EBV-infected cells.

## Results

### BCR pathway genes are targeted by EBNA2 and EBNA3 proteins

Previous EBNA2 and EBNA3A, 3B and 3C (from now on referred to as EBNA3) ChIP-sequencing data from EBV-infected Burkitt lymphoma cells generated in our laboratory [10] was used to identify the cellular genes and pathways that may be regulated by the binding of these transcription factors. Three different criteria were used to link significant binding sites (MACS < 10^−7^) for EBNA2 and EBNA3 to the proximal or distal cellular genes they may regulate. The first criterion identified cellular genes that had any significant EBNA binding site with 2 kB of their transcription start sites (promoter-proximal). The second criterion identified genes that were the closest (irrespective of distance) to the top most significant EBNA binding sites: it was reasoned that the most significant binding sites were more likely to represent functional gene regulatory elements. For EBNA3 the top 300 most significant binding sites were used for this analysis. For EBNA2, where a higher number of highly significant binding sites had identical *P*-values, binding sites with a *P*-value of 10^−310^ or lower (526 sites) were used for analysis. The third criterion was less stringent and simply identified target genes that were closest to any significant binding site (MACS < 10^−7^). Pathway analysis (DAVID (https://david.ncifcrf.gov/) was performed using these sets of gene lists to identify the cellular pathways targeted by the EBNAs. Our aim was to identify key genes in pathways that may be important for B cell immortalisation and growth deregulation by EBV for follow up analysis to confirm their regulation and explore the mechanisms involved. For genes with promoter-proximal binding sites for EBNA2 or EBNA3 proteins and for genes that were closest to the top most significant EBNA2 binding sites, we identified the B cell receptor (BCR) signalling pathway as the only significantly enriched pathway (Bonferonni < 0.01, Table 1). Analysis of the genes closest to the top EBNA3 binding sites did not show significant enrichment for any cellular pathway. Combining the genes identified in the BCR pathway from the first two criteria returned 25 unique genes as potential targets for EBNA2 and EBNA3 proteins (*IFITM1, NFKBIA, CD72, RASGRP3, CD22, PIK3AP1, NFATC4, INPP5D, PIK3R1, NFATC1, PIK3R2, SYK, PTPN6, CR2, VAV3, LYN, PIK3CD, VAV2, PRKCB, MAPK1, CD19, CD81, PLCG2, CD79B, CD79A*) (Table 1). Pathway analysis using genes identified from criteria three (closest to any significant EBNA binding site) also identified the BCR signalling pathway as a significantly enriched pathway for both EBNA2 and EBNA3 binding sites (Table 1). For EBNA2, BCR signalling was the second most enriched pathway, with the first being T-cell receptor signalling, although many genes in these two pathways overlap. For the EBNA3s, BCR signalling was also the second most enriched pathway (Table 1), with JAK-STAT signalling the most enriched pathway, although again many genes featured in both pathways (Table 1). Taken together, all three criteria identified 63 unique genes in the BCR signalling pathway that could represent genes regulated by EBNA2 or the EBNA3 family of proteins. Previous analysis also identified the BCR signalling pathway as the most enriched pathway for genes associated with super-enhancers bound by EBV transcription factors in EBV-infected cells [48]. Some genes in our lists had already been identified as genes regulated by EBNA2, 3A, 3B or 3C in transcriptome studies, but this regulation had not been further confirmed or investigated.

**Table 1.** Pathway analysis

Signalling pathway	*P*-value	Genes	Bonferroni	Benjamini	FDR
*EBNA2 promoter-proximal*					
B cell receptor signalling pathway	1.39 × 10^−5^	PTPN6, CD19, RASGRP3, NFKBIA, CD22, CD79B, NFATC4, CD79A, CD72, PIK3R1, SYK	0.001759	0.001759	0.016079
*EBNA3 promoter-proximal*					
B cell receptor signalling pathway	1.87 × 10^−6^	PTPN6, VAV3, NFKBIA, CD72, PRKCB, MAPK1, CD19, RASGRP3, CD22, NFATC4, CD79B, CD79A, PIK3R1, SYK	2.82 × 10^−4^	2.82 × 10^−4^	2.23 × 10^−3^
*EBNA2 top significant*					
B cell receptor signalling pathway	2.61 × 10^−9^	PTPN6, CR2, IFITM1, LYN, PIK3CD, VAV2, CD19, CD81, PLCG2, CD22, CD79B, PIK3AP1, NFATC4, CD79A, INPP5D, NFATC1, PIK3R2	3.50 × 10^−7^	3.50 × 10^−7^	3.06 × 10^−6^
*EBNA2 closest*					
B cell receptor signalling pathway	1.95 × 10^−12^	HRAS, NFKB1, BTK, FOS, PIK3CA, PIK3AP1, SYK, AKT2, PIK3CG, BCL10, LYN, PIK3CB, RELA, LOC646626, PIK3CD, PRKCB, CARD11, MAPK1, JUN, CD81, IFITM1, NFKBIE, GRB2, PPP3R1, NFKBIA, CD72, LOC407835, KRAS, RASGRP3, DAPP1, RAC2, SOS1, RAC1, PPP3CB, NFAT5, PPP3CC, CD22, NFATC4, PIK3R5, PPP3CA, INPP5D, PIK3R3, NFATC2, NFATC3, PIK3R1, NFATC1, PIK3R2, BLNK, PTPN6, VAV3, CR2, MAP2K1, MAP2K2, MALT1, VAV2, VAV1, CD19, GSK3B, PLCG2, CD79B, CD79A, IKBKB	3.37 × 10^−10^	1.87 × 10^−10^	2.42 × 10^−9^
*EBNA3 closest*					
B cell receptor signalling pathway	3.96 × 10^−10^	NFKBIA, CD72, LOC407835, FOS, KRAS, RASGRP3, DAPP1, RAC2, SOS1, PPP3CC, CD22, NFATC4, PIK3AP1, PPP3CA, INPP5D, PIK3R3, NFATC3, AKT3, PIK3R1, NFATC1, BLNK, SYK, PIK3CG, PTPN6, BCL10, VAV3, CR2, LYN, PIK3CB, MAP2K2, LOC646626, VAV2, PRKCB, CARD11, MAPK1, CD19, CD81, PLCG2, CD79B, CD79A, IKBKB	7.40 × 10^−8^	3.70 × 10^−8^	4.90 × 10^−7^

Results of DAVID pathway analysis using gene lists identified from EBNA2 and EBNA3s ChIP-seq data from Mutu III cells [10]. Enrichment of the BCR signalling pathway was identified for genes with promoter-proximal (within 2Kb) EBNA2 and EBNA3 binding sites, genes closest to the top most significant EBNA2 binding site, or genes close to any significant EBNA2 or EBNA3 binding site.

### EBNA2 represses transcription of many BCR pathway target genes in an EBV-infected LCL

**Figure 1.**
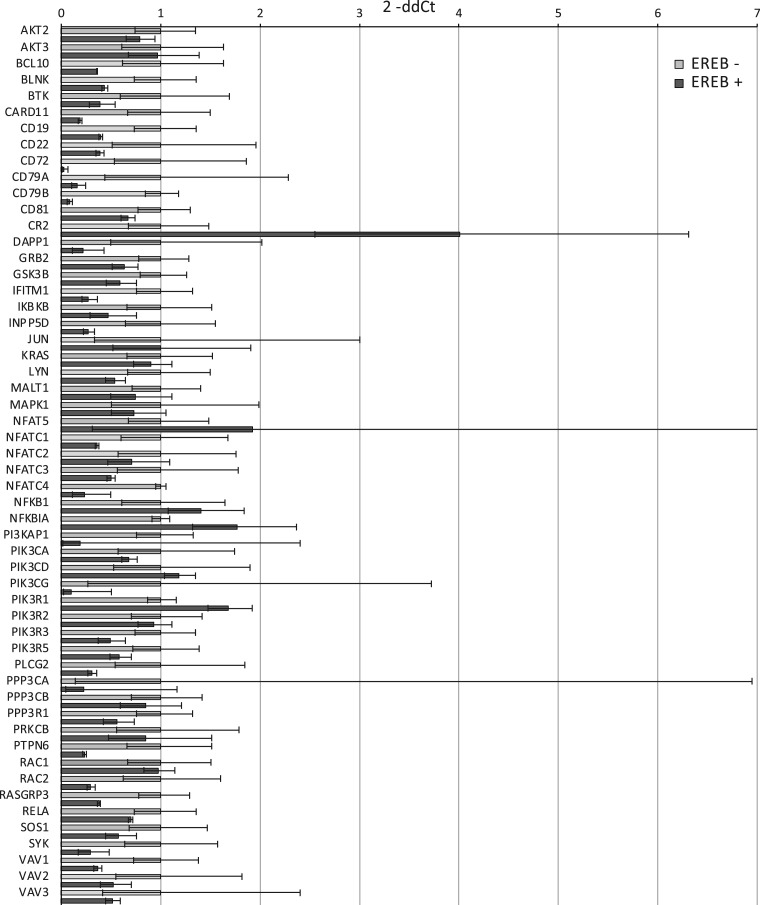
Taqman array card analysis of BCR pathway gene expression in ER-EB 2.5 cells. cDNA was generated from two biological replicate samples from ER-EB 2.5 cells (which express a conditionally active oestrogen receptor-EBNA2 fusion protein) maintained in the absence of β-estradiol for 4 days and with 1 µM β-estradiol was re-added for 17 h. Gene expression was normalised to the housekeeping gene, *GUSB*. Normalised gene expression (dCt) was calculated by deducting the Ct value of *GUS*B for each sample from the Ct value of the gene in question. *P*-values cited in the text derive from a two-sample *t*-test on the dCt-values for +EBNA2 (+ β-estradiol) relative to −EBNA2 (− β-estradiol). Relative quantitation was used to generate average ddCt values for +EBNA2 relative to −EBNA2 and standard deviation (ddCt sd). Fold change was derived as 2^−ddCt^ and plotted for each gene +EBNA2 (dark grey bars) relative to −EBNA2 (light grey bars) samples (error bars show 2^−ddCt±sd^). *FOS* and *BCL6* were below detectable levels in one or more of our samples so are not shown.

To determine whether the BCR pathway genes linked to EBNA2 binding sites were regulated by EBNA2, we examined gene expression in an EBV-infected B cell line that expresses a conditionally active oestrogen receptor-EBNA2 fusion protein (ER-EB 2.5) [49]. cDNA generated from duplicate RNA samples extracted from cells incubated without β-estradiol and with β-estradiol re-added (to activate EBNA2) was analysed using TaqMan array cards pre-loaded with primer sets for 56 of the potential EBNA2 and EBNA3 BCR pathway target genes plus four control genes (*GAPDH*, *RPLP0*, *GUSB* and *HPRT1*) for normalisation. All of these 56 genes with the exception of *AKT3* were identified as potential EBNA2 targets (Table 1). We did not include LOC407835 (a *MAPKK2* pseudogene) or LOC646626 (an uncharacterised noncoding RNA) in our array card gene set. *NFKBIE*, *HRAS*, *MAP2K1*, *MAP2K2*, *PIK3CB* and *PPP3CC* were also excluded due to the presence of very small EBNA2 or EBNA3 binding sites. Interestingly, although best characterised as an activator, our data demonstrated that EBNA2 repressed the majority of target genes analysed (Figure 1). Two genes that were significantly up-regulated included two known activation targets of EBNA2, *CR2* [50] (dCt *P*-value 0.018) and *PIK3R1* [42] (dCt *P*-value 0.011), providing good validation for our analysis (Figure 1). In general, our results corroborated previous observations of EBNA2-mediated repression of *CD72*, *CD79A*, *CD79B*, *NFATC1*, *PIK3CG*, *PLCG2*, *RASGRP3* and *VAV1* when conditionally active EBNA2 was expressed in an EBV-negative B cell line (BJAB) [17]. Our analysis also highlighted potential new repressed target genes including *NFATC4* (which showed a trend to repression) and *IFITM1* (dCt *P*-value 0.003). Our data indicate that for some BCR pathway genes, the direction of regulation can be cell-type dependent, as we have previously observed for EBNA-regulated genes [10,29]. Many genes repressed by EBNA2 in ER-EB 2.5 cells (*CD22*, *PTPN6*, *BLNK*, *CD19*, *LYN* and *PPP3CA*) are up-regulated in other cell contexts [17,43]. Overall, we found that all 11 BCR pathway genes with a promoter-proximal EBNA2 binding sites (Table 1) showed some evidence of repression. In fact, in the ER-EB 2.5 LCL background, our data indicate that the majority of the 56 BCR pathway genes linked to EBNA2 binding sites are repressed to some extent by EBNA2.

### EBNA3 proteins repress some BCR pathway genes with promoter-proximal binding peaks

**Figure 2.**
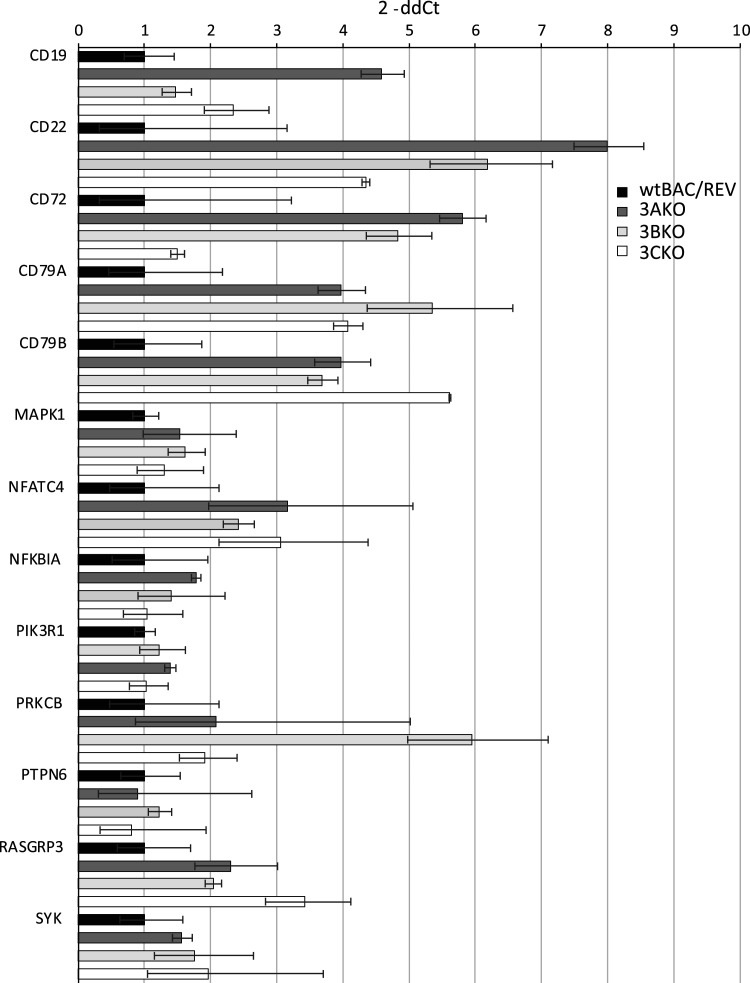
Taqman array card analysis of expression of EBNA3 promoter-proximal BCR pathway genes in EBV-infected BL31 cells. Analysis as in Figure 1 for BL31 wt Bac and revertant lines (wt Bac2, wt Bac3, 3A rev, 3B rev, 3C rev) (black bars), BL31 3A KO (dark grey bars), BL31 3B KO (light grey bars), BL31 3C KO (white bars). Samples for BL31 KOs were derived from two separate cell line clones. Data for *VAV3* are not shown as it was undetectable in one or more of the cell lines.

Regulation of BCR pathway genes by EBNA3 proteins was also examined using the BCR gene Taqman array cards with RNA samples from two different cell backgrounds. The first set of cell lines analysed were EBV-negative Burkitt lymphoma cells (BL31) infected with either wild-type recombinant EBV (wtBAC), individual EBNA3 knock-out EBVs (BL31 3AKO, BL31 3BKO, BL31 3CKO) or the respective revertant viruses where the deleted gene was reintroduced (BL31 3A rev2, BL31 3B rev2.2, BL31 3C rev2) [46]. Of the 56 BCR genes on the array cards, 36 genes were identified as potential EBNA3 target genes from ChIP-seq analysis (Table 1). Fourteen of these genes had a promoter-proximal binding peak for an EBNA3 protein (Table 1). Taqman array card data from the two wtBAC cell lines (BL31 wtBAC2 and BL31 wtBAC3) were combined with data from the four revertant lines, since they all express the full wild-type complement of EBV latent genes. These data were compared with combined data from individual pairs of EBNA3A, 3B and 3C KO cell lines for the 14 genes with promoter-proximal binding sites (Figure 2). We found that *CD22*, *CD79A* and *CD79B* showed a trend towards higher expression in all three KO cell lines, suggesting that EBNA3A, 3B and 3C play a role in their repression (Figure 2). *CD72* displayed a trend towards higher expression in the EBNA3A and EBNA3B KO cell lines and *PRKCB* in EBNA3B KO cell lines, indicating EBNA3-mediated repression as reported previously [46]. We also detected repression of an additional four genes by EBNA3A not previously reported: *CD19* (dCt *P*-value 0.0013), *NFATC4* (dCt *P*-value 0.015) and *RASGRP3* (dCt *P*-value 0.003). Overall, we found that 8/14 genes with promoter-proximal EBNA3 binding sites show some evidence of repression by at least one EBNA3 family member.

Of the remaining genes on the Taqman array cards, 22 had distal EBNA3 binding sites (Table 1)*.* Of these, *CR2* was not detected in one or more sample. Out of the 17 remaining distal genes, *NFATC1* and *PIK3AP1* were repressed by EBNA3A, 3B and 3C consistent with previously reported EBNA3 repression [46] with *FOS* also showing repression by all EBNA3s (Supplementary Figure S1). We also identified two new repressed target genes *NFATC3* (by EBNA3A; dCt *P*-value 0.015) and *KRAS* (by EBNA3B and EBNA3A; dCt *P*-values 0.008). We detected up-regulation of two genes; *PIK3R3* by EBNA3A (dCt *P*-value 0.04) and *VAV2* by EBNA3B (dCt *P*-value 0.006) consistent with previous reports of activation by the EBNA3s (Supplementary Figure S1) [46]. Overall, 7/22 distal genes showed some evidence of regulation by an EBNA3 protein. Surprisingly, although our data support the previous detection of *NFATC2* as an EBNA3 repressed gene in BL31 cells [46], this gene was assigned to an EBNA2 not EBNA3 binding site (see later) in our gene identification process although it showed little EBNA2 regulation in ER-EB 2.5 cells (Supplementary Figure S1, Figure 1 and Table 1).

The second set of cell lines analysed for evidence of EBNA3 regulation of BCR pathway genes were LCLs. Initially we examined LCLs generated by infection with either wild-type EBV or knock-out/mutant viruses lacking either EBNA3A or 3B. Of the 14 genes with promoter-proximal EBNA3 binding sites, one (*NFATC4*) was not detected in one or more sample. Of the remaining 13 genes, only *CD72* showed evidence of regulation (activation) by EBNA3A, despite this gene being a gene repressed by EBNA3 proteins in BL31 cells (Supplementary Figure S2). Of the 16/22 detectable genes with distal EBNA3 binding sites, *AKT3* showed a trend towards repression by EBNA3A as previously reported [45] and *BLNK* (dCt *P*-value 0.02) and *LYN* (dCt *P*-value 0.02) showed a low level of statistically significant activation by EBNA3Anot previously reported (Supplementary Figure S3) [45]. In EBNA3B KO LCLs, of the 12 detected genes with promoter-proximal EBNA3 binding sites, only *CD22* showed a trend towards repression by EBNA3B supporting our data from BL31 cells (Supplementary Figure S4). Of the 16 genes with distal EBNA3 binding sites that with detectable expression, none showed any evidence of regulation by EBNA3B in LCLs (Supplementary Figure S5).

To examine EBNA3C gene regulation in an LCL background we used an LCL expressing a hydroxy-tamoxifen (HT) responsive oestrogen receptor-EBNA3C fusion protein [51]. Samples were taken from cells grown in the presence of HT (EBNA3C active), in the absence of HT (EBNA3C inactive) and then with HT added back (EBNA3C reactivated). Due to space on the array, we were only able to analyse single samples for trends in regulation. Of the 13/14 detectable genes with promoter-proximal EBNA 3 binding sites, only *CD72* and *PIK3R1* showed good evidence of regulation, with both genes showing some repression by EBNA3C (Supplementary Figure S6) consistent with published data from BL31 cells [46], although our own analysis did not confirm this (Figure 2). Of the 22 genes with distal EBNA3 binding sites (Table 1), *CR2*, *PIK3AP1*, *PIK3CG*, *PIK3R3*, *RAC2*, *SOS1* and *VAV2* showed evidence of repression by EBNA3C where *BLNK* and *NFATC1* showed evidence of activation (Supplementary Figure S7). Although detected as potential EBNA2 and not EBNA3 target genes, *AKT2*, *PIK3R2*, *PPP3CB* and *VAV1* also showed some evidence of repression (Supplementary Figure S7).

In summary, across all cell lines examined, we found evidence of repression by EBNA2 and at least one EBNA3 protein for *BLNK*, *CD22*, *CD72*, *CD79A*, *CD79B*, *NFATC1*, *PIK3CG* and *RASGRP3*. In addition, we found evidence of repression of *PIK3AP1* and activation of *PIK3R3* by EBNA3 proteins in both the BL and an LCL background. Overall, our data indicate that EBNA2 and EBNA3 repress the expression of many genes in the BCR pathway, including the central BCR components *CD79A* and *CD79B* involved in antigen-mediated signalling, the inhibitory BCR co-receptors *CD22* and *CD72* and key signalling adaptor proteins, kinase subunits and transcription factors involved in the downstream calcium, MAPK and PI3K-Akt signalling pathways.

### EBNA2 represses *CD79B* by interfering with EBF-1 activation

**Figure 3.**
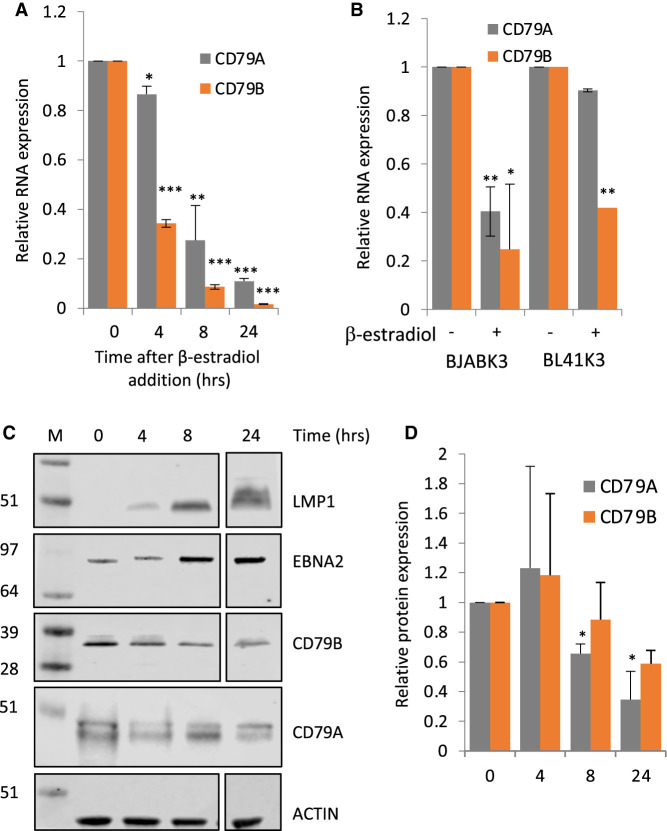
CD79A and CD79B RNA and protein expression analysis. (**A**) QPCR analysis of *CD79A* and *CD79B* transcript mRNA expression in EREB 2.5 cells untreated (0) or treated with β-estradiol for 4, 8 and 24 . Signals are normalised to GAPDH and show mean expression levels relative to (−ER) ± standard deviation of two QPCR experiments. *P*-values were calculated relative to the (−ER) sample and significance indicated as follows; * (*P*-value > 0.05), ** (*P*-value ≤ 0.05), *** (*P*-value ≤ 0.01). The experiment is representative of three independent experiments. (**B**) QPCR analysis of *CD79A* and *CD79B* mRNA expression in BJABK3 and BL41K3 cells treated with 1 μM β-estradiol for 24 h. Signals are normalised to GAPDH and show mean expression levels relative to (−ER) ± standard deviation of two QPCR experiments. *P*-values were calculated relative to the (−ER) sample and significance indicated as in (**A**). (**C**) Representtaive western blot analysis of LMP1, EBNA2, CD79A and CD79B protein levels in ER-EB 2.5 cells untreated (0) or treated with β-estradiol for 4, 8 and 24 h. Actin was used as a loading control. LMP1, EBNA2, CD79B and ACTIN blots have been cut to remove a 17 h point not available for the CD79A blot for consistency of presentation. Quantification of CD79A and CD79B protein expression is shown for three independent experiments ± standard deviation for CD79A and two independent experiments ± standard deviation for CD79B. CD79A and CD79B signals on western blots were normalised to actin and are expressed relative to ER-EB 2.5 (−ER).

Since repression of *CD79A* and *CD79B* is likely to affect all downstream BCR signalling, our initial studies investigated the mechanism of repression of these genes. We focused on EBNA2-mediated down-regulation of *CD79A* and *CD79B* since EBNA2-mediated repression mechanisms remain poorly understood and repression of these genes has been detected in a range of B cell backgrounds. *CD79A* and *CD79B* were previously reported as significantly down-regulated by EBNA2 in the B cell lymphoma cell line BJAB [17], but in our Taqman analysis using duplicate ER-EB 2.5 RNA samples, repression of these genes did not reach statistical significance. We therefore carried out further analysis to confirm their down-regulation in ER-EB 2.5 cells using samples taken up to 24 h after re-addition of β-estradiol to activate EBNA2. We found statistically significant down-regulation of both genes over this timecourse (Figure 3A). *CD79B* mRNA levels decreased more rapidly and to a greater extent than *CD79A* mRNA levels following EBNA2 activation (Figure 3A). The decreased expression of *CD79A* and *CD79B* was also confirmed at the protein level (Figure 3C,D), although protein levels varied between experiments at the 4 h timepoint and did not fall to the same level as mRNA expression likely due to protein stability over the timeframe used. As a control for EBNA2 activation, we also examined expression of the EBNA2 activated EBV target gene LMP1. As expected, LMP1 protein levels increased on β-estradiol addition (Figure 3C). Although binding of β-estradiol results in EBNA2 activation through translocation of (ER)-EBNA2 to the nucleus, some increased expression of EBNA2 is evident on β-estradiol addition due to EBNA2-responsiveness of the promoter that drives fusion protein expression, as reported previously [49]. We also observed the previously reported shift in apparent molecular mass of (ER)-EBNA2 on β-estradiol addition [49]. To further validate the regulation of *CD79A* and *CD79B*, we also examined their expression in previously studied BJAB and BL41 (EBV-negative BL) cell lines expressing (ER)-EBNA2 [17]. Consistent with published data, we detected a reduction in *CD79B* mRNA expression 24 h after EBNA2 activation in BJAB and BL41 cell lines (Figure 3B) [17]. Again, consistent with published data we only observed a reduction in *CD79A* expression on EBNA2 activation in BJAB cells and not BL41 cells (Figure 3B) [17]. Up-regulation of expression of the EBNA2 target gene *CR2* confirmed the β-estradiol-mediated activation of EBNA2 in BJAB and BL41 cell lines (Supplementary Figure S8).

**Figure 4.**
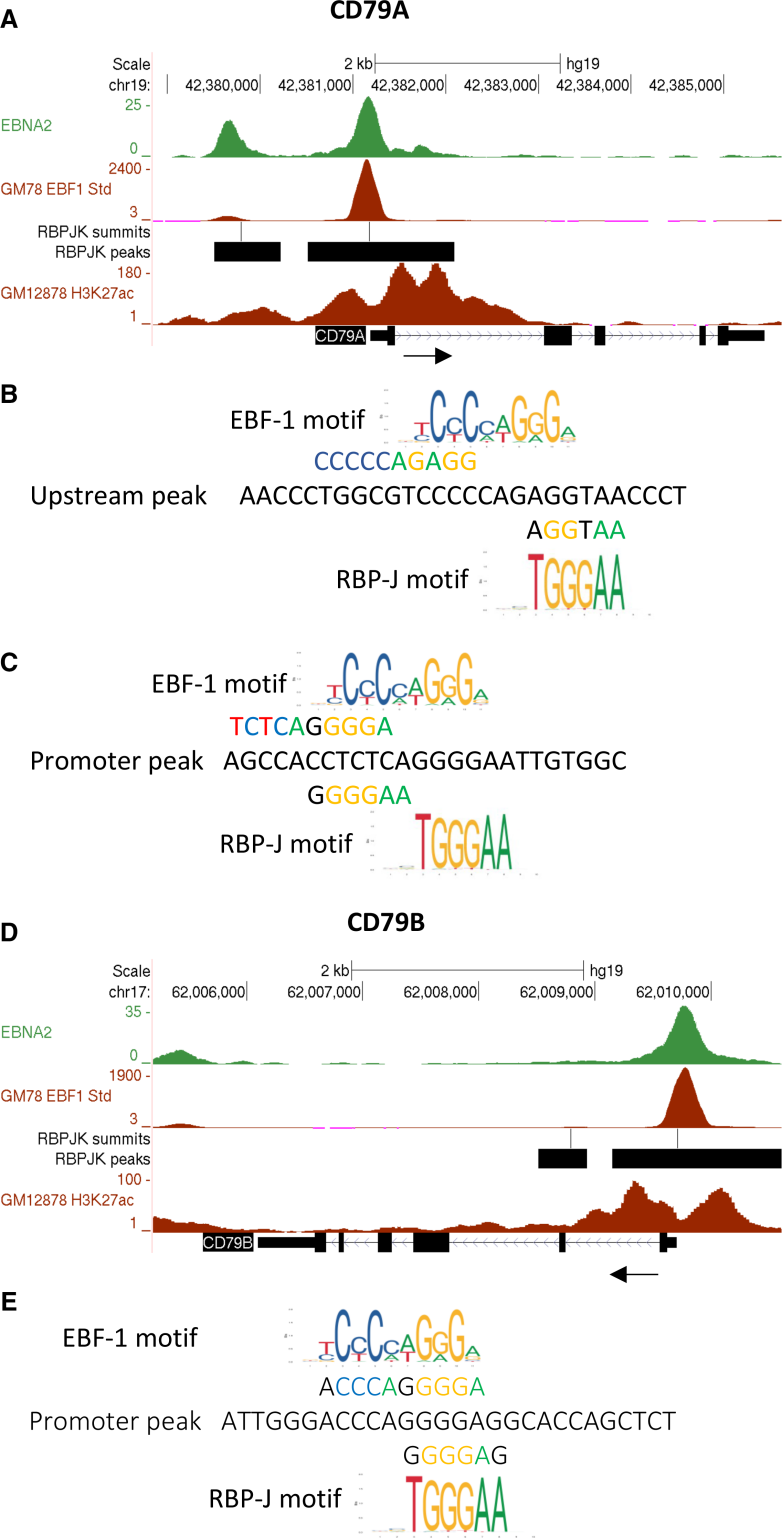
EBNA2 binding sites at *CD79A* and *CD79B*. (**A**) The number of EBNA2 sequencing reads from immunoprecipitated Mutu III and GM12878 DNA are plotted per million background-subtracted total reads and aligned with the human genome at *CD79A*. The black arrow indicates the direction of gene transcription. GM12878 EBF-1 and H3K27 acetylation ChIP-seq data from ENCODE are also shown at the bottom of the panel. RBP-J binding sites identified by ChIP-seq [9] using the IB4 LCL are shown as their called MACS peaks (black bars) and summits of peaks (lines). (**B**) Transcription factor binding motifs at the upstream promoter-proximal peak at *CD79A* showing overlapping EBF-1 and potential RBP-J motifs. (**C**) EBF-1 and potential RBP-J overlapping motifs at the *CD79A* proximal promoter peak. (**D**) EBNA2 binding at *CD79B*. (**E**) Transcription factor binding motifs at the *CD79B* binding site.

To investigate the mechanism of EBNA2-mediated repression of *CD79A* and *CD79B*, we examined the location of EBNA2 binding sites at *CD79B* and *CD79A* mapped by our original ChIP-sequencing analysis in the EBV positive Mutu III BL cell line [10]. EBNA2 binds at the *CD79A* TSS and at an additional site ∼1.5 kb upstream (Figure 4A). At *CD79B* EBNA2 binds a single site close to the TSS (Figure 4D). Consistent with the promoter function of these regions, publicly available acetylated H3K27 ChIP-sequencing data from the EBV-immortalised LCL GM12878 (ENCODE) shows peaks of H3K27 acetylation (Figure 4A,D). Since EBNA2 does not bind DNA directly, we next examined the binding of known EBNA2 binding partners and regulators of *CD79A* and *CD79B* transcription at these promoter regions [52–54]. We found that EBNA2 binding peaks coincided with binding peaks for EBF-1 and RBP-J (Figure 4A,D). Transcription factor binding motif analysis at the *CD79A* and *CD79B* EBNA2 binding sites identified overlapping sequences with similarity to EBF-1 and RBP-J consensus motifs (Figure 4C,E). The absence of repressive chromatin marks at *CD79A* and *CD79B* promoters in EBV-infected cells (not shown) and the known dependency of these genes on EBF-1 activity [55], led us to hypothesise that EBNA2 may reduce their transcription by interfering with gene activation by EBF-1. This could be mediated through competitive binding of an EBNA2–RBP-J complex or by direct binding of EBNA2 to EBF-1.

**Figure 5.**
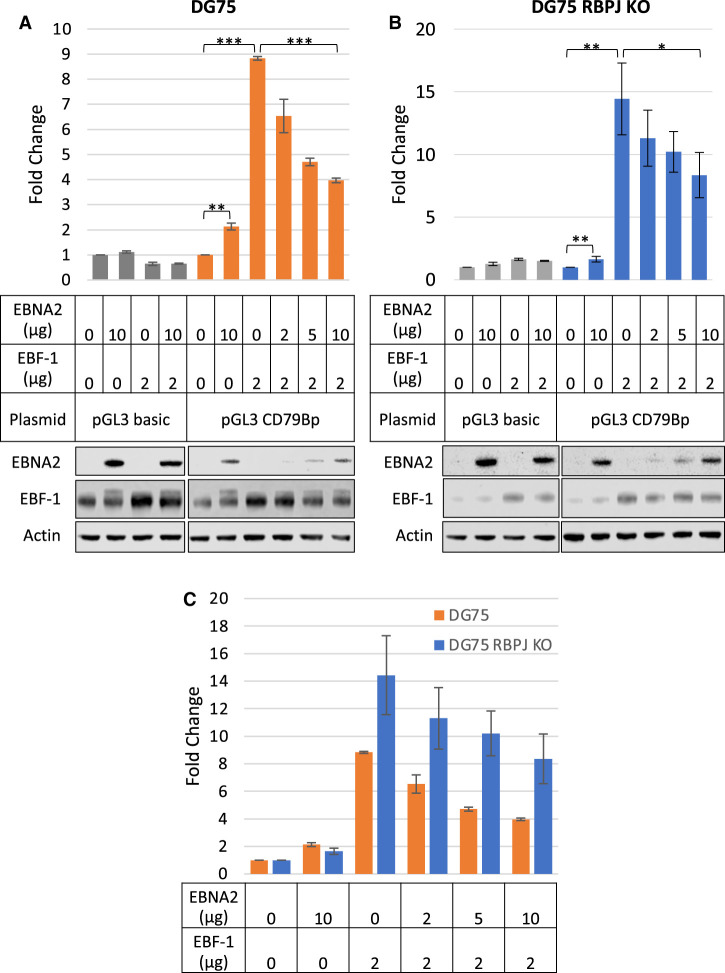
Luciferase assays examining the effect of EBNA2 and EBF-1 on *CD79B* promoter activity. (**A**) DG75 cells were transiently transfected with 2 μg of the control vector pGL3 Basic or pGL3_CD79Bp reporter constructs in the presence or absence of an EBNA2 expressing construct (pSG52A) and/or an EBF-1 expressing construct (pCMV-SPORT62-EBF-1). Cells were co-transfected with a Renilla control plasmid pRL-CMV (0.5 μg). Firefly luciferase signals were normalised to Renilla luciferase signals. Results show the mean ± standard deviation of two independent experiments and are expressed relative to signal in the absence of EBNA2 or EBF-1. Lower panels show western blot analysis of EBNA2 and EBF-1 expression in transfected cells. Actin was used as a loading control. *T*-tests were performed and significance indicated as follows; * (*P*-value > 0.05), ** (*P*-value ≤ 0.05), *** (*P*-value ≤ 0.01). (**B**) DG75 RBP-J KO cells were transfected as in (**A**). (**C**) A direct comparison between promoter activity in DG75 and DG75 KO cells (data from (**A**) and (**B**)).

To test this hypothesis, we generated a luciferase reporter construct containing the EBNA2 binding site at the *CD79B* promoter, since repression of *CD79B* was most robust and reproducible in different cell backgrounds and EBNA2 bound a single site at high level (Figure 4D). We transfected the EBV-negative B cell line DG75 with this luciferase reporter construct in the presence and absence of an EBF-1 expressing construct and increasing quantities of an EBNA2 expressing construct. EBF-1 activated the *CD79B* promoter over 8-fold at the plasmid concentration used in this assay (Figure 5A). We also observed a small activating effect of EBNA2 (2-fold) on the *CD79B* promoter in the luciferase assay (Figure 5A). However, in the presence of EBF-1, increasing amounts of EBNA2 led to decreased *CD79B* promoter activity supporting the observations of a repressive effect of EBNA2 on the promoter when the promoter is bound by EBF-1 as it would be in EBF-1-positive B cells (Figure 5A).

To determine whether the repressive effect of EBNA2 on EBF-1 activation of the *CD79B* promoter was dependent on RBP-J, we repeated the luciferase reporter assays in a DG75 RBP-J (CBF-1) knock-out cell line. We observed an increase in transcriptional activation by EBF-1 in RBP-J KO cells (14-fold compared with 8-fold), consistent with RBP-J acting as a repressor of *CD79B* transcription through inhibition of EBF-1 DNA binding [52] (Figure 5B,C). The absence of RBPJ had no effect on the 2-fold activation of the *CD79B* promoter by EBNA2 indicating that the small activating effect of EBNA2 is not dependent on its binding via RBP-J (Figure 5). Consistent with our results in wild-type DG75 cells, we found that in RBP-J KO cells the activation of *CD79B* by EBF-1 was reduced in the presence of increasing amounts of EBNA2 expressing plasmid (Figure 5B,C). Our data, therefore, indicate that the repression of EBF-1 activation by EBNA2 at *CD79B* is not dependent on RBP-J and is therefore unlikely to be the result of competitive binding of an EBNA2–RBP-J complex to the overlapping RBP-J/EBF-1 binding site in the *CD79B* promoter.

**Figure 6.**
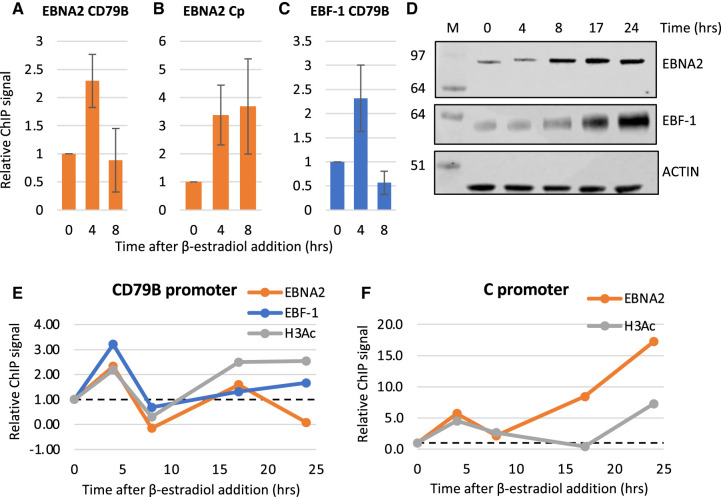
ChIP-QPCR analysis of EBNA2 and EBF-1 binding. (**A**) ChIP-QPCR for EBNA2 at the *CD79B* promoter in untreated EREB 2.5 cells or cells treated with β-estradiol for 4 or 8 h. Results show mean ± standard deviation from three independent ChIP experiments with percentage input signals after subtraction of IgG antibody controls expressed relative to the signal in the absence of β-estradiol at time 0. (**B**) ChIP-QPCR for EBNA2 binding at the EBV C promoter as in (**A**). (**C**) ChIP-QPCR for EBF-1 binding at the *CD79B* promoter as in (**A**). (**D**) Western blot analysis for ER-EBNA2 and endogenous EBF-1 expression in ER-EB 2.5 cells (±β-estradiol). Actin was used a loading control. (**E**) Representative ChIP results (from three independent experiments) for EBNA2, EBF-1 and H3acetylation at the *CD79B* promoter for a longer ER-EB 2.5 induction time course. Analysis as in (**A**). (**F**) Representative ChIP results (from three independent experiments) for EBNA2, EBF-1 and H3acetylation at the EBV C promoter for a longer ER-EB 2.5 induction time course.

We next investigated the effects of EBNA2 on EBF-1 association with the *CD79B* promoter in an EBV-infected cell line using ChIP-QPCR. We examined EBNA2 and EBF-1 binding over a timecourse of induction of EBNA2 activity in ER-EB 2.5 cells through the addition of β-estradiol. Over an initial 8 h timecourse, where we observed robust repression of *CD79B* mRNA expression (Figure 3A), EBNA2 binding to the *CD79B* promoter and the control EBV C promoter was detected at 4 h (Figure 6A,B). At this timepoint, increased EBF-1 binding was also detected indicating that EBNA2 binding may enhance binding of EBF-1 (Figure 6C). However, 8 h post EBNA2 activation, EBNA2 binding at the *CD79B* promoter fell to near background levels (despite some increased EBNA2 expression) (Figure 6A,D) and we observed a corresponding drop in EBF-1 binding to below the original level of binding observed prior to EBNA2 activation (Figure 6C). This reduction in binding of both proteins at *CD79B* correlates with a 10-fold reduction in *CD79B* mRNA expression 8 h after β-estradiol addition (Figure 3B). Since EBNA2 binds to EBF-1, it is possible that the formation of the EBNA2–EBF-1 complex leads to its reduced association with the promoter. To explore this further we carried out ChIP-QPCR analyses over a longer timecourse (Figure 6D–F). These longer analyses have to be interpreted in the context of increased expression of EBF-1 in ER-EB 2.5 cells 8–24 h after β-estradiol addition; EBF-1 is an EBV EBNA1 activated gene [56], in addition to the increase in EBNA2 levels observed from 8 h (Figure 6B). Although the kinetics of transcription factor binding at the *CD79B* promoter changed slightly in different timecourses, we observed a consistent pattern of an initial increase in EBNA2 and EBF-1 binding, followed by a reduction in binding and then apparent recovery of EBF-1 binding, but not EBNA2 binding (Figure 6A,C,E). It is likely that the restored EBF-1 binding to levels slightly above those detected at the *CD79B* promoter prior to β-estradiol addition is simply a consequence of increased EBF-1 expression at these later time points. Interestingly, histone H3 acetylation levels increased at 4 h, dropped and then recovered despite gene expression being repressed (Figure 6E). In contrast at the viral C promoter, EBNA2 showed reduced binding at 8 h in some experiments but binding then recovered and increased over the time course, accompanied by later increases in histone H3 acetylation levels (Figure 6B,F). It is clear that events at the *CD79B* promoter are complex and hard to track by population average ChIP-QPCR experiments and in the context of changing transcription factor expression. Taken together, our data raise the possibility that EBNA2 may bind to the promoter transiently and cause some initial destabilisation of EBF-1 binding. This may be sufficient to cause reduced transcription.

### EBNA2 and EBNA3 proteins target NFAT genes for repression through intragenic and promoter-proximal binding sites

We next examined how other BCR pathway genes are targeted by EBNA2 and EBNA3 proteins. Our ChIP-sequencing analysis identified all five members of the NFAT family, the effector transcription factors of the calcium signalling pathway, as potential EBNA2 and EBNA3 target genes. *NFATC1* (NFAT2) was identified as the closest gene to a top significant EBNA2 peak and a significant EBNA3 binding peak (Table 1 and Supplementary Figure S9). Three clusters of 8 large EBNA2 binding peaks are located within the *NFATC1* gene, with the most significant EBNA2 binding site located 73 kb downstream from the *NFATC1* TSS. EBNA3 binding across the *NFATC1* locus is weak, but two small intragenic EBNA3 peaks that are distinct from the EBNA2 binding sites were identified as significant (MACS < 10^−7^) (Supplementary Figure S9). We found good evidence of repression of *NFATC1* expression by EBNA2 in ER-EB2.5 cells and by all three EBNA3 proteins in BL31 cells (Figure 1 and Supplementary Figure S1). This is consistent with previous reports of EBNA2-mediated repression of *NFATC1* in BJAB cells [17] and EBNA3A and EBNA3C-mediated repression of NFATC1 in the same BL31 cell lines [46].

*NFATC2* was identified as a gene closest to a significant EBNA2 binding peak and contains multiple significant EBNA2 binding peaks throughout the gene (Supplementary Figure S9). Surprisingly, this gene was not identified as a potential EBNA3 target gene, despite the presence of a large EBNA3 binding peak that coincides with the location of an EBNA2 binding peak ∼81 kb downstream from the TSS (Supplementary Figure S9), and two smaller EBNA3 binding peaks further downstream. These EBNA3 peaks were assigned to MIR3194, located in an *NFATC2* intron in our gene assignment analysis. Consistent with the presence of EBNA3 binding peaks, *NFATC2* was confirmed as an EBNA3 repressed target gene in our analysis (Supplementary Figure S1), although we could not confirm EBNA2-mediated repression of *NFATC2* in ER-EB2.5 cells.

*NFATC3* (NFAT4) was identified as the closest gene to significant EBNA2 and EBNA3 binding peaks. EBNA3 bound at one site within the gene and at a site upstream in the *DUS2* gene where an EBNA2 peak was also present (Supplementary Figure S9). *NFATC3* showed evidence of repression by EBNA3 proteins in BL31 cells (Supplementary Figure S1) but was not convincingly repressed by EBNA2 in ER-EB 2.5 cells. *NFATC4* (NFAT3) is targeted by coincident promoter-proximal binding of EBNA2 and EBNA3, with EBNA2 binding at the highest level observed across all NFAT genes (Supplementary Figure S9). Consistent with this, *NFATC4* was repressed by EBNA2 in ER-EB 2.5 cells and showed evidence of repression by EBNA3 proteins in BL31 cells (Supplementary Figure S1). *NFATC3* and *NFATC4* have not previously been identified as EBNA2 or EBNA3 regulated genes. *NFAT5* was identified as an EBNA2 target gene and has an EBNA2 binding site at the TSS, although it was not identified as a promoter-proximal target gene (Supplementary Figure S9). We did not, however, obtain any evidence for EBNA2 regulation of NFAT5 as its expression was highly variable in our samples (Figure 1). There were no EBNA3 binding peaks in and around *NFAT5* and *NFAT5* was not convincingly regulated by EBNA3 and has not been reported as an EBNA3 target gene.

Taken together, our data indicate that *NFATC1* and *NFATC4* can be repressed by EBNA2. These genes have the largest EBNA2 binding peaks of the NFATs examined, so regulation appears to correlate with the level of EBNA2 binding. *NFATC1*, *NFATC2*, *NFATC3* and *NFATC4* can be repressed by EBNA3 proteins, with NFATC2 showing the largest change in expression, which again correlates with the presence of a large EBNA3 binding peak at *NFATC2*. The extent of binding near NFAT genes, therefore, predicts regulation by the EBNAs better than the presence of binding peaks alone.

### EBNA2 and EBNA3 proteins attenuate NFAT activation

**Figure 7.**
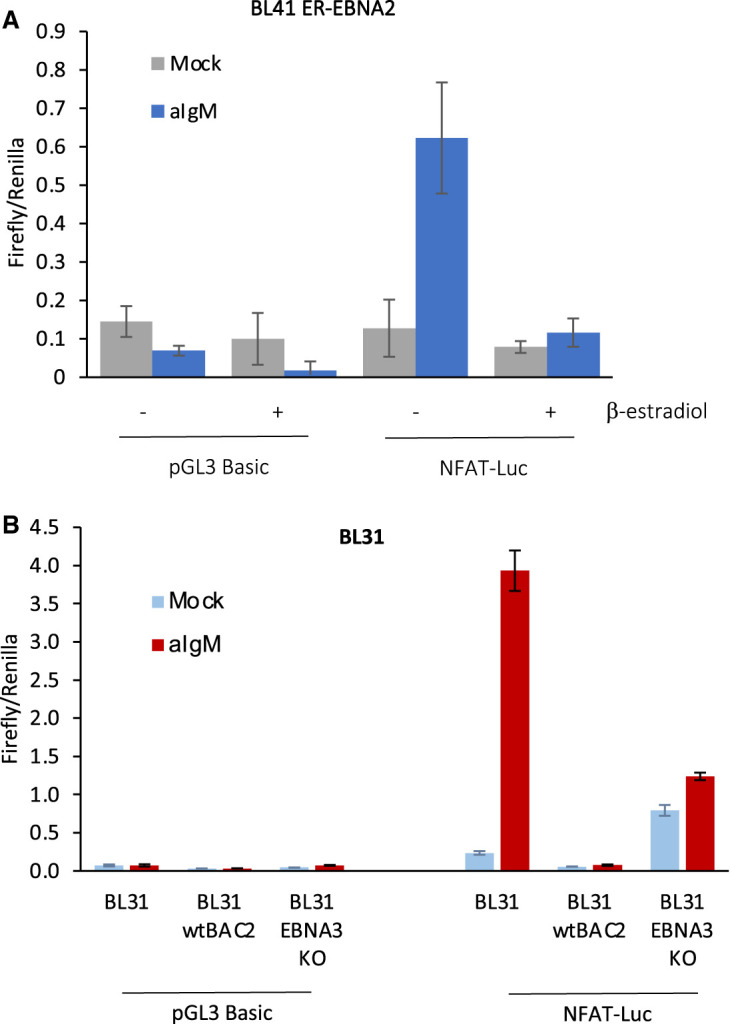
EBNA2 and EBNA3 proteins attenuate NFAT activity. (**A**) EBV-negative BL cells expressing a conditionally active (ER)-EBNA2 fusion protein (BL41K3) were transfected with 5 μg of empty plasmid (pGL3 basic) or the pGL3_NFAT luciferase reporter and 2.5 μg of control plasmid pRL-TK. Cells were incubated for 24 h in the absence or presence of β-estradiol followed by an 18 h incubation ± anti-IgM and analysed for luciferase activity. Firefly signals were normalised to Renilla luciferase signals from the co-transfected control plasmid pRL-TK. Results show the mean ± standard deviation from two independent experiments analysed in duplicate. (**B**) EBV-negative BL31 cells or cells infected with wild-type EBV Bacmid (BL31 wt Bac2) or EBNA3A, 3B and 3C knock-out EBV (BL31 EBNA3 KO) were transfected and incubated with anti-IgM as above.

We next examined whether the regulation of BCR pathway genes (including *CD79A*, *CD79B*, the key adaptors *LYN* and *SYK* and the *NFAT* family of genes) by EBNA2 and EBNA3 proteins affected BCR signalling through the Ca^2+^ arm in B cells using NFAT activity as a read out. For these experiments, we used Burkitt lymphoma cell lines where BCR activation could be induced by cross-linking with anti-IgM antibodies. We first examined the impact of EBNA2 on BCR signalling using EBV-negative BL41 cells expressing the conditionally active (ER)-EBNA2 fusion protein. Cells were transiently transfected with an NFAT luciferase reporter promoter plasmid containing 3 NFAT binding motifs upstream of a luciferase promoter [57] in the absence or presence of ß-estradiol and then treated (or mock treated) with anti-IgM antibodies. In the absence of active EBNA2 (minus ß-estradiol) we detected a 4.8-fold increase in NFAT reporter activity following anti-IgM treatment (Figure 7A). However, when EBNA2 was activated by ß-estradiol, anti-IgM treatment only induced a 1.5-fold increase in NFAT activity indicating that EBNA2 attenuates BCR-induced NFAT activation. We next examined the effects of expression of the EBNA3 proteins on BCR signalling using the BL31 cell line series. We found that in EBV-negative BL31 cells anti-IgM treatment resulted in a 16-fold induction of NFAT reporter activity (Figure 7B). In contrast, in BL31 cells infected with wt EBV, we observed a 4-fold reduction in basal NFAT activity and a complete absence of induction of NFAT activity following anti-IgM treatment. Interestingly, in BL31 cells infected with EBNA3 knock-out virus, basal NFAT activity was restored to a level above those in EBV-negative BL31 cells (Figure 7B). However, we observed only a partial restoration of the response to BCR activation with anti-IgM treatment resulting in only a 1.6-fold increase in NFAT reporter activity. This indicates that EBNA3 proteins can suppress NFAT activity in unstimulated BL31 cells and play a partial role in attenuating NFAT activity in response to BCR activation. It is likely that other EBV-encoded gene products (including EBNA2) play a greater role in the attenuation of the response to BCR cross-linking in EBV uninfected BL31 cells (Figure 7B).

In summary our data indicate that EBNA2 and EBNA3 proteins can suppress the basal or active BCR signalling that culminates in NFAT activation. The effects of EBNA2 and EBNA3 proteins on NFAT activity could stem from reduced expression of the BCR components CD79B or CD79A combined with repression of other calcium pathway genes including the NFATs themselves. In BL41 cells, EBNA2 represses *CD79B*, but not *CD79A* (Figure 3C and [17]). We also found that the key calcium signalling component phospholipase C gamma 2 (*PLC-γ2*) and *NFATC1* were not significantly repressed at the mRNA level in our BL41 cell line samples (Supplementary Figure S10), although *NFATC1* (but not the other EBNA2 target gene *NFATC4*) was identified as a repressed gene by Maier et al. [17]. The adaptors *SYK* and *LYN* are also not reported as EBNA2 repressed genes in BL41 cells [17]. It is therefore likely that the effects of EBNA2 on NFAT activation in BL41 cells mainly stem from down-regulation of *CD79B* expression resulting in reduced signalling from the membrane. In BL31 cells, the impact of EBNA3 proteins on basal and induced NFAT activity could be mediated through their repression of *CD79A* and *CD79B* in addition to multiple NFATs (*NFATC1*, *NFATC2*, *NFATC3* and *NFATC4*).

### EBNA2 and EBNA3 proteins repress the PI3K-Akt pathway through the inactivation of Akt

We next investigated the impact of EBNA2 and EBNA3 proteins on the PI3K-Akt pathway since in addition to *CD79A* and *CD79B* multiple genes involved in this pathway are regulated targets. For EBNA2 our data showed the majority of target genes involved in PI3K-Akt signalling were repressed (including *CD19*, *SYK*, *LYN*, *PIK3R3*, *PIK3R5*) consistent with suppression of BCR signalling, although repression of the inhibitor *INPP5D* and increased expression of *PIK3R1* expression may be expected to elicit positive effects (Figure 1). *CD19* was repressed by EBNA3A and *PIK3AP1* was repressed by all EBNA3s in BL31 cells (Figure 2 and Supplementary Figure S1) and *AKT3*, *BLNK* and *LYN* were repressed by EBNA3A in LCLs (Supplementary Figure S3) indicating a potential suppressive role for the EBNA3s in PI3K-Akt signalling.

**Figure 8.**
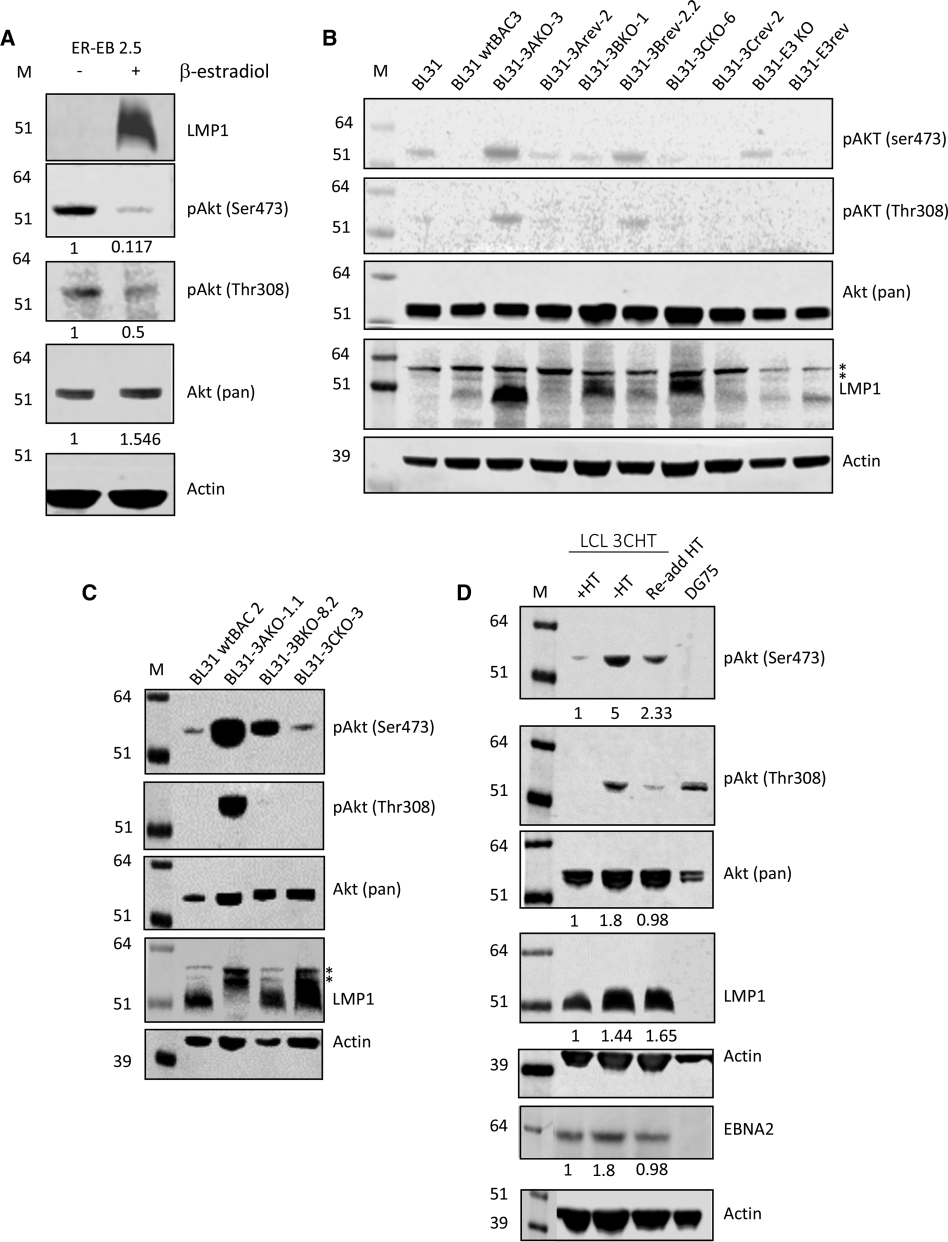
Western blot analysis of Akt phosphorylation in different EBV-infected cell lines. (**A**) Analysis of activated Akt phosphorylation at Ser473 and Thr308 in ER-EB2.5 cells in the absence of β-estradiol (−) or 17 h after β-estradiol re-addition (+). Blots were also probed with a pan Akt antibody to detect total Akt levels and anti-LMP1 antibodies. Actin was used as a loading control. Numbers under panels show quantification of these representative western blots with signals for each antibody normalised to actin and expressed relative to ER-EB 2.5 (−β-estradiol). (**B**) Western blot analysis in the BL31 cell line series infected with either wild-type recombinant EBV (BL31 wtBAC3, BL31 wtBAC2), EBNA3A knock-out EBV (BL31 3AKO-3AΔ, BL31-3AKO-1.1), EBNA3B knock-out EBV (BL31-3BKO-1, BL31-3BKO-8.2), EBNA3C knock-out EBV (BL31-3CKO-6, BL31-3CKO-3), EBNA3 knock-out EBV (BL31-E3KO) or with their respective revertant viruses (BL31-3A rev-2, BL31-3B rev-2.2, BL31-3C rev-2 and BL31-E3 rev). Non-specific bands (*). (**C**) Western blot analysis of additional BL31 knock-out cell lines (**D**) Western blot analysis of a conditional LCL expressing an EBNA3C-HT fusion protein cultured in the absence of HT for 21 days and then with HT re-added for 10 days. Numbers under panels show quantification of these representative western blots with signals for each antibody normalised to actin and expressed relative to −HT.

To examine the overall impact of EBNA2 and EBNA3 proteins on Akt signalling, we measured the levels of total Akt and the levels of active Akt (phosphorylated on Thr308 and Ser473) by western blotting [58]. Expression of LMP1 was also monitored as LMP1 can increase the basal activity of Akt through phosphorylation on Ser473 [59]. We found that the levels of the Ser473 and Thr308 phosphorylated forms of Akt were reduced in ER-EB 2.5 cells in the presence of active EBNA2 (+ ß-estradiol) (Figure 8A). This was observed despite a small increase in the levels of total Akt and in the presence of induced LMP1 expression due to the activation of the LMP1 promoter by EBNA2 (Figure 8A). Our data, therefore, indicate that EBNA2 suppresses Akt activation.

We next examined the effect of EBNA3 proteins using the BL31 cell line series. We observed that levels of Ser473 and Thr308 phosphorylated forms of Akt were reduced in BL31 cells infected with wild-type EBV (BL31 wtBAC3 and wtBAC2) and most revertant viruses, with variation in levels observed across the cell lines examined (Figure 8B,C). Consistently, in BL31 cells infected with EBNA3A KO or EBNA3 KO viruses, active phospho-Akt levels were equivalent or higher than in uninfected BL31 cells indicating that EBNA3A suppresses Akt activation (Figure 8B,C). Although phospho-Akt levels were higher in one 3B KO cell line clone examined, phospho-Thr308 was not increased (Figure 8C). There was also no consistent impact of loss of EBNA3C expression on active Akt levels in BL31 cells (Figure 8B,C). However, in LCLs, phosphorylation of Akt on Ser473 and Thr308 was reduced in cells with active EBNA3C (Figure 8D) and increased on inactivation of EBNA3C. Although an increase in total Akt protein was also observed on inactivation of EBNA3C, the increase in phospho-Ser473 Akt was 3-fold even when adjusted for this (Figure 8D). This indicates that EBNA3C can suppress Akt activation in an LCL context. Together, our data indicate that EBNA2, EBNA3A and EBNA3C can suppress PI3K-Akt signalling, with the effect of EBNA3C being cell context dependent.

## Discussion

Our study demonstrates that multiple genes in signalling pathways downstream of the BCR and the BCR itself are subject to down-regulation by EBNA2 and EBNA3 proteins, with the binding of gene regulatory elements and transcription repression by these EBNAs likely playing an important role. EBV manipulates various aspects of B cell activation to induce cell proliferation and differentiation [60]. Evidence for BCR signalling regulation by these EBNAs provides a new dimension through which EBV-encoded gene products can manipulate BCR-mediated survival and growth signals in infected cells. Like LMP1 and BHRF 1–2 miRNAs, EBNA2 and 3 proteins suppress BCR signalling. Suppressing BCR signal transduction is important for maintaining viral latency in infected B cells since activation of the BCR triggers plasma cell differentiation and a switch from latency to lytic replication [60]. Consistently, active NFATC1 and NFATC2 can induce lytic EBV gene expression in EBV positive Burkitt lymphoma cells [61]. By supressing both NFAT and PI3K signalling, these EBNAs are therefore likely to work alongside other EBV latent gene products to maintain viral latency in EBV-infected cells. Since PI3K, Akt and the NFATs can be activated through signalling initiated at a range of receptors such as integrins, receptor tyrosine kinases and cytokine receptors, it is possible that the effects we observe of EBNA2 and EBNA3 proteins may also be the result of their impact on other signalling pathways. For example, EBNA3C is known to down-regulate genes in the integrin receptor signalling pathway [29].

We identified large promoter-proximal EBNA2 binding sites at *CD79A* and *CD79B.* Since *CD79A* and *CD79B* form the signal transducing subunit of the BCR receptor and are expressed in virtually all immature and mature B cells [62], this places regulatory control by the EBNAs right at the start of the BCR signalling cascade*.* We confirmed that EBNA2 was a negative regulator of *CD79A* and *CD79B* in both LCL and lymphoma cell backgrounds and we also showed reduced CD79A and CD79B protein levels in response to EBNA2 activation in LCLs. The chromatin landscape surrounding the promotor proximal EBNA2 binding sites at *CD79A* and *CD79B* corresponded with regions of H3K27 acetylation in LCLs indicating that the EBNA2 bound-promoter regions retained active chromatin marks in cells that express EBNA2 despite their reduced expression. Transcription factor binding site analysis of the *CD79A* and *CD79B* EBNA2 binding sites revealed overlapping binding sites for EBF-1 and RPB-J, although the EBF-1 binding sites were a better match than the RPB-J sites. Luciferase reporter assays using the EBNA2 bound-promoter regions of *CD79B*, in EBV-negative DG75 cells and DG75-RBP-J-KO cells showed that EBNA2 alone was able to activate the promoter, independently of RBP-J. This is interesting as EBNA2 activation of *CD79B* is not observed *in vivo*. This discrepancy may be because other repressive factors present at the *CD79B* promoter preclude EBNA2-mediated activation of these genes *in vivo* and the chromatin context at the promoter in EBV-infected is also likely to differ from that in transfected plasmids. Nonetheless, we observed that co-expression of EBNA2 and EBF-1 led to a decrease in activation of the *CD79B* promoter by EBF-1, independently of RBP-J. This is consistent with a previous study that identified *CD79B* as an RBP-J independent EBNA2 repressed gene and showed EBNA2 and EBF-1 can bind *in vitro* [5]. Based on our data, and the fact that EBF-1 can act as an EBNA2 binding partner we hypothesised that EBNA2 binds EBF-1 hinders its ability to activate transcription of *CD79B*.

When we further explored the interplay between EBF-1 and EBNA2 at the *CD79B* promoter *in vivo* using ChIP-QPCR we found that EBNA2 and EBF-1 both bound the *CD79B* promoter and their binding patterns follow similar kinetics, with an initial increase then reduction in binding of both factors until 24 hrs when EBNA2 binding reduced and EBF-1 binding seemed to stabilise, despite continued repression of *CD79B* mRNA levels. There is clearly a complex series of events happening at the promoter that are hard to disentangle using population average ChIP assays, but we propose that EBNA2 is destabilising EBF-1 binding at *CD79B* and interfering with EBF-1 mediated transcriptional activation. This is consistent with EBNA2 functioning as a passive rather than active repressor.

Focusing on other genes downstream of the BCR, we identified multiple members of the NFAT family (*NFATC1*, *NFATC2*, *NFATC3* and *NFATC4*), key effectors of the calcium signalling arm of the pathway, as repressed target genes of both EBNA2 and EBNA3s. Although we did not explore the mechanisms of transcriptional repression of the NFATs, multiple binding peaks were present in promoter-proximal and more distal intragenic locations, with the extent of binding of the EBNAs being a good indicator of extent of regulation. Thus, regulation at the beginning and end of the BCR pathway by the EBNAs can contribute to BCR signalling suppression.

Importantly, using NFAT activation of a reporter plasmid as a readout of NFAT activity, we showed that EBNA2 repressed BCR-mediated NFAT activation in Burkitt lymphoma cells and EBNA3-mediated suppression of mostly basal NFAT activity. This links gene suppression by the EBNAs to a physiological impact on BCR signalling. In some cell contexts, these effects may largely be due to effects on *CD79B*, where in others combined effects on *CD79A* and *CD79B* plus the *NFATs* and other signalling pathway components may also contribute. Cell-type specific differences in gene regulation by the EBNAs has been reported previously [10,29] and may impact on the extent of BCR signalling suppression. Effects of EBV on BCR signalling also differs through the different EBV latency stages due to the decrease in the number of EBV latent proteins expressed as B cells differentiate. EBNA2 and EBNA3 proteins are only expressed in initial EBV growth transformed lymphoblasts for example.

We also examined EBNA2 and EBNA3 repression of the PI3K-Akt signalling pathway by examining the effect on EBNA2 and EBNA3 proteins on activation of Akt via phosphorylation on Ser473 and Thr308 residues. The net effect of EBNA2, EBNA3A and EBNA3C proteins in both BL and LCLs appears to lead to reduced active phosphorylation of Akt demonstrating a potential negative impact on the pathway. As LMP1 has been shown to activate the PI3K-Akt pathway by phosphorylation of Akt on Ser473 [59], this positive and negative modulation of BCR signalling by EBV latent proteins could be a strategy employed by the virus to promote the growth-survival balance of EBV-infected cells.

Overall, the repression of BCR genes and multiple genes from the calcium and PI3K-Akt signalling pathways by EBNA2 and EBNA3 proteins may be a strategy used by EBV to fine tune cell growth and survival signalling, complementing the activities of LMP1 and LMP2A and the BHRF 1–2 miRNAs in certain cellular contexts. Further work will be required to fully understand the mechanism of both EBNA2 and EBNA3-mediated gene repression of these gene targets, in particular the role of EBNA2 as a passive pressor and whether EBNA3s employ similar polycomb repressor-mediated repression mechanisms to suppress their BCR pathway target genes. Given that both EBNA2 and EBNA3 proteins can remodel three-dimensional chromatin structure to modulate enhancer–promoter interactions, it will also be interesting to determine whether any distal binding sites around the BCR signalling genes identified interact with gene promoters and indeed whether any further longer-range EBNA binding sites are involved in gene regulation.

## Methods

### Cell lines

All cell lines were maintained in RPMI-1640 media (Gibco, U.K.) supplemented with 10% Fetal Bovine Serum, Penicillin and Streptomycin (Gibco, U.K.). The EBV-negative Burkitt lymphoma cell line BL31 infected with wild-type recombinant EBV bacmids or EBNA3A, 3B and 3C-exon 2 knock-out or EBNA 3A, 3B and 3C combined knock-out viruses (herein named respectively as 3AKO, 3BKO, 3CKO and E3KO) and their revertant bacmids (3Arev, 3Brev, 3Crev and E3 rev) were provided by Prof M. Allday [26]. Cells were cultured with appropriate supplements and selection as described previously [26].

LCLs established by infecting primary B cells with either wild-type EBV (LCL3A wt) or mutant virus lacking the coding sequence for EBNA3A (LCL 3A mutB) were provided by Prof Kempkes [45]. LCL EBNA3B KO, wt BAC and EBNA3B revertant cell lines established by infecting peripheral blood leukocytes (PBLs) from donor 2 (D2) were provided by Prof M. Allday and maintained as described previously [45,46]. The LCL 3CHT was also provided by Prof M. Allday and has been described previously [51]. For 4-HT (Sigma, U.K.) withdrawal and add back experiments, cells were maintained in the presence of 400 nM of 4-HT for 25 days, HT was then washed off and cells were cultured in media without HT for 21 days. HT was re-added as required and cells were cultured for a further 10 days.

The EBV-immortalised LCL (ER-EB 2.5) expresses a conditionally active oestrogen receptor (ER)-EBNA2 fusion protein and was cultured in the presence of 1 μM β-estradiol (Sigma). For inactivation and reactivation of EBNA2, cells were maintained in the absence of β-estradiol for 4 days and 1 μM β-estradiol was re-added for either 4, 8, 17 or 24 h prior to cell harvest. The EBV-negative Burkitt lymphoma cell lines BL41K3 and BJABK3) (kindly provided by Prof B. Kempkes) (Kempkes et al. [49]) also express a conditionally active oestrogen receptor (ER)-EBNA2 fusion protein were cultured in the absence of β-estradiol and 1 μM β-estradiol added to activate EBNA2 as required. The EBV-negative Burkitt Lymphoma cell line DG75 derived from a biopsy taken in 1975 [63]. The DG75 CBF-1KO cell line is a derivative of DG75 cells where the CBF-1/RBP-J gene has been inactivated by homologous recombination [64] was a gift from Prof Kempkes.

### Plasmid construction

To create pGL3 CD79B, a 0.94 kb fragment of the *CD79B* promoter (∼–765 to +176 relative to the predicted transcription start site) was amplified from genomic DNA extracted from a B cell line using primers to create *NheI* and *SacI* sites. The digested PCR product was then cloned into the *Nhel* and *SacI* sites of pGL3-Basic (Promega).

### SDS–PAGE and western blotting

SDS–PAGE and western blotting was performed as described previously [65] using the following primary antibodies: anti-LMP1 (mouse monoclonal, CS1-4), anti-Akt pan (rabbit monoclonal, CST #C67E7), anti-Akt phospho T308 (rabbit monoclonal, CST #13038), anti-Akt phospho S473 (rabbit monoclonal, CST #4060), anti-EBNA2 (mouse monoclonal, PE2), anti-CD79A (rabbit polyclonal, CST #3351), anti-CD79B (rabbit polyclonal, Abcam #175399), anti-EBF-1 (mouse monoclonal, Santa Cruz, SC137065), anti-Actin (rabbit polyclonal, Sigma #A2066). Blots were visualised and the signal was quantified on a Li-COR imager.

### RNA isolation and gene expression analysis using TaqMan array cards

Total RNA was isolated from BL31 cell lines, LCL stable cell lines and ER-EB 2.5 cells using Tri Reagent (Sigma) according to the manufacturer's instructions. cDNA was synthesised from 1 μg of RNA using the ImProm-II Reverse Transcription System (Promega). Ninety-six-well Custom TaqMan array cards (Thermo Fisher) were designed and ordered and arrived pre-loaded with gene expression assays for selected BCR signalling genes associated with EBNA2 or EBNA3 binding sites. Array cards also contained four reference gene expression assays (GAPDH, GUSB, RPLP0 and HPRT1) for relative quantification. Two-hundred nanograms of cDNA was added to TaqMan Universal PCR Master Mix (2×) with UNG in a final volume of 100 μl. Samples were subsequently loaded into each fill reservoir through ports on TaqMan array cards. Real-time PCR was performed on Applied Biosystems 7900HT Real-time PCR system with a TaqMan Array Microfluidic Card Thermal Cycling block using cycling conditions (50°C/2min, 95°C/10min, then (95°C/15 s, 60^o^C/1 min)) for 40 cycles provided by the manufacturer. The data were analysed with the cloud suite software Symphoni (Life Technologies) using the Relative Quantification Module.

### QPCR

One microgram RNA was used to prepare cDNA using the ImProm-II Reverse Transcription System with random primers (Promega). QPCR was carried out in duplicate using the standard curve absolute quantification method on an Applied Biosystems StepOne Plus Real-time PCR system as described previously using cDNA specific primers for *CD79A*, *CD79B*, *NFATC1*, *NFATC2*, *PLCγ2*, *CD21*, *Cp* and *GAPDH* (Supplementary Table S1)*,* with the GAPDH signal used as the normalisation control [10].

### Chromatin immunoprecipitation

For the ER-EB 2.5 cell line, cells were cultured in medium without β-estradiol for 4 days. After 4 days of withdrawal, a control sample (time 0) was taken and β-estradiol (1 μM) was then added and chromatin prepared 4, 8, 17 and 24 h later. Chromatin was prepared from 1 × 10^7^ cross-linked cells as described previously [10]. For chromatin immunoprecipitation, EBNA2 was precipitated using 8 µg of PE2 (mouse monoclonal, gift from M. Rowe) and EBF-1 was precipitated using 2 µg of mouse monoclonal EBF-1 antibody (Santa Cruz, SC137065). H3 acetylation ChIP was performed using 5 µg of rabbit polyclonal Acetyl-Histone H3 antibody (MerckMillipore, 06-599). For QPCR analysis, both input and IP samples were analysed using primers detailed in Supplementary Table S1. ChIP efficiencies were calculated as the percent of input DNA immunoprecipitated and the mouse monoclonal IgG antibody (Santa Cruz, SC2025) was used as a control. Control IgG percentage input values were subtracted from target protein percentage input values.

### Luciferase reporter assays

For the CD79B reporter activity assays, 1 × 10^7^ DG75 and DG75-RBP-J KO cells were transfected using the Bio-Rad Gen Pulser II Electroporation system as described previously [65–67]. Cells were transfected with either 2 μg of pGL3 Basic or pGL3 CD79Bp in the presence or absence of an EBNA2 expressing construct (pSG52A) and an EBF-1 expressing construct (pCMV-SPORT62-EBF-1). pRL-CMV (0.5 μg) was included as a transfection control. For NFAT activity assays, 1 × 10^6^ BL41K3 or BL31 cells were transfected using the Neon Transfection System (Invitrogen). Cells were transfected with 5 μg of pGL3 NFAT reporter containing 3 NFAT binding motifs upstream of a luciferase promoter (gift from Prof A. Sinclair [57]) and pRL-TK (2.5 μg) as a transfection control, using a 100 μl Neon tip and 1 pulse of 1300 V for 30 msec. 1 μM β-estradiol was added 5 h after transfection of BL31K3 cells to activate EBNA2 and for all cell lines 10 μg/ml anti-Human IgM (Merck, I0759) was added 24 h after transfection to cross-link the BCR. Cells were harvested after 48 h and a luciferase assay was performed using the Dual-Luciferase Reporter Assay System (Promega). The firefly luciferase signal was adjusted for transfection efficiency using the Renilla luciferase signal from the control plasmids pRL-CMV or pRL-TK.

## Data Availability

All supporting data are included within the main article and its supplementary files.

## Open Access

Open access for this article was enabled by the participation of University of Sussex in an all-inclusive *Read & Publish* agreement with Portland Press and the Biochemical Society under a transformative agreement with JISC.

## Abbreviations

BCR: B cell receptor
BLNK: B cell linker kinase
EBNA: EBV nuclear antigens
EBV: Epstein–Barr virus
HT: hydroxy-tamoxifen
ITAMs: immunoreceptor tyrosine-based activation motifs
LCLs: lymphoblastoid cell lines
LMP: latent membrane proteins
