# Supplementary material for: Regulation of B cell receptor signalling by Epstein–Barr virus nuclear antigens
Source: Biochem J. 2022 Dec 7;479(23):2395–417. doi: 10.1042/BCJ20220417 (PMC9788576; doi:10.1042/BCJ20220417)
Supplement: Supplementary Material [file BCJ-479-2395-s1.pdf]

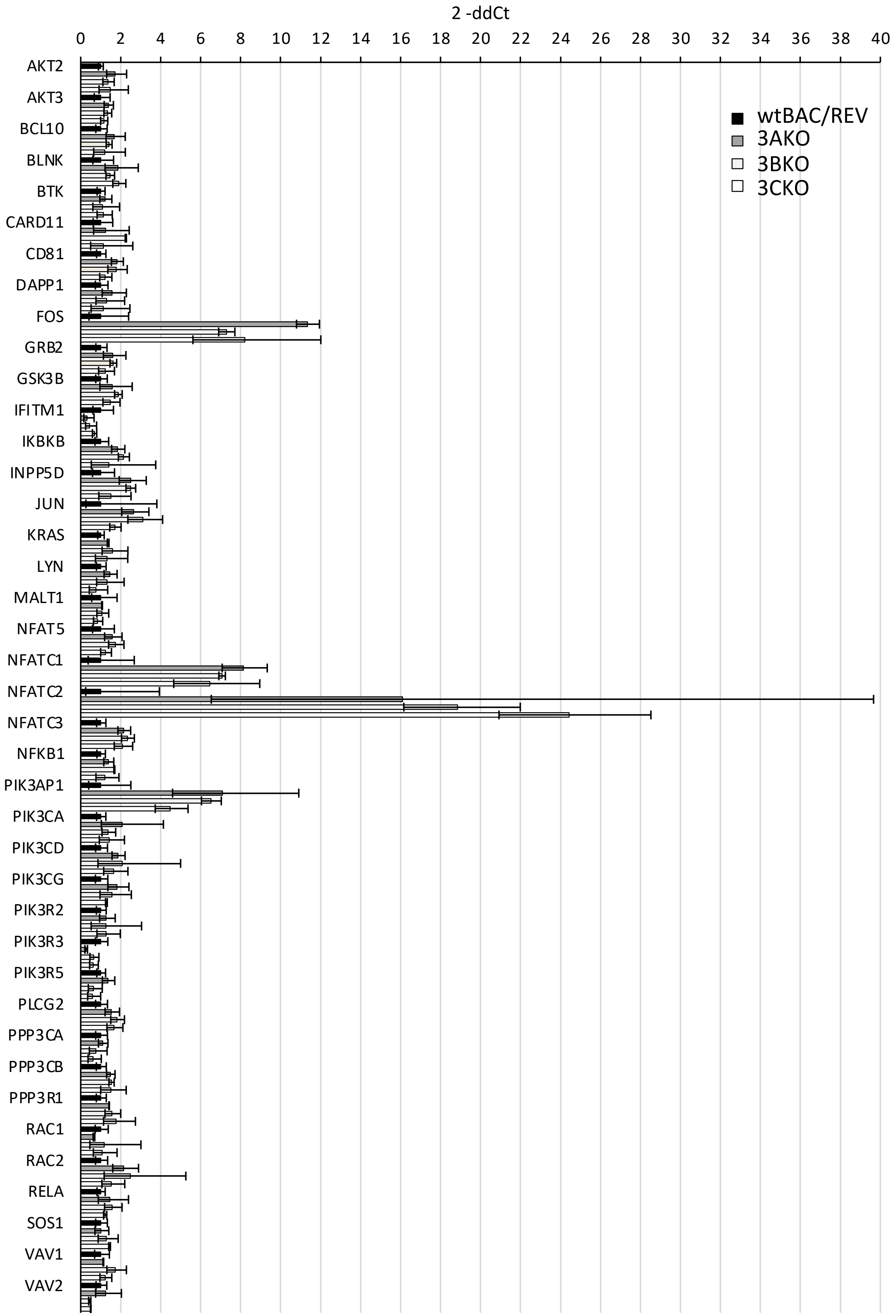

Supplementary Figure 1

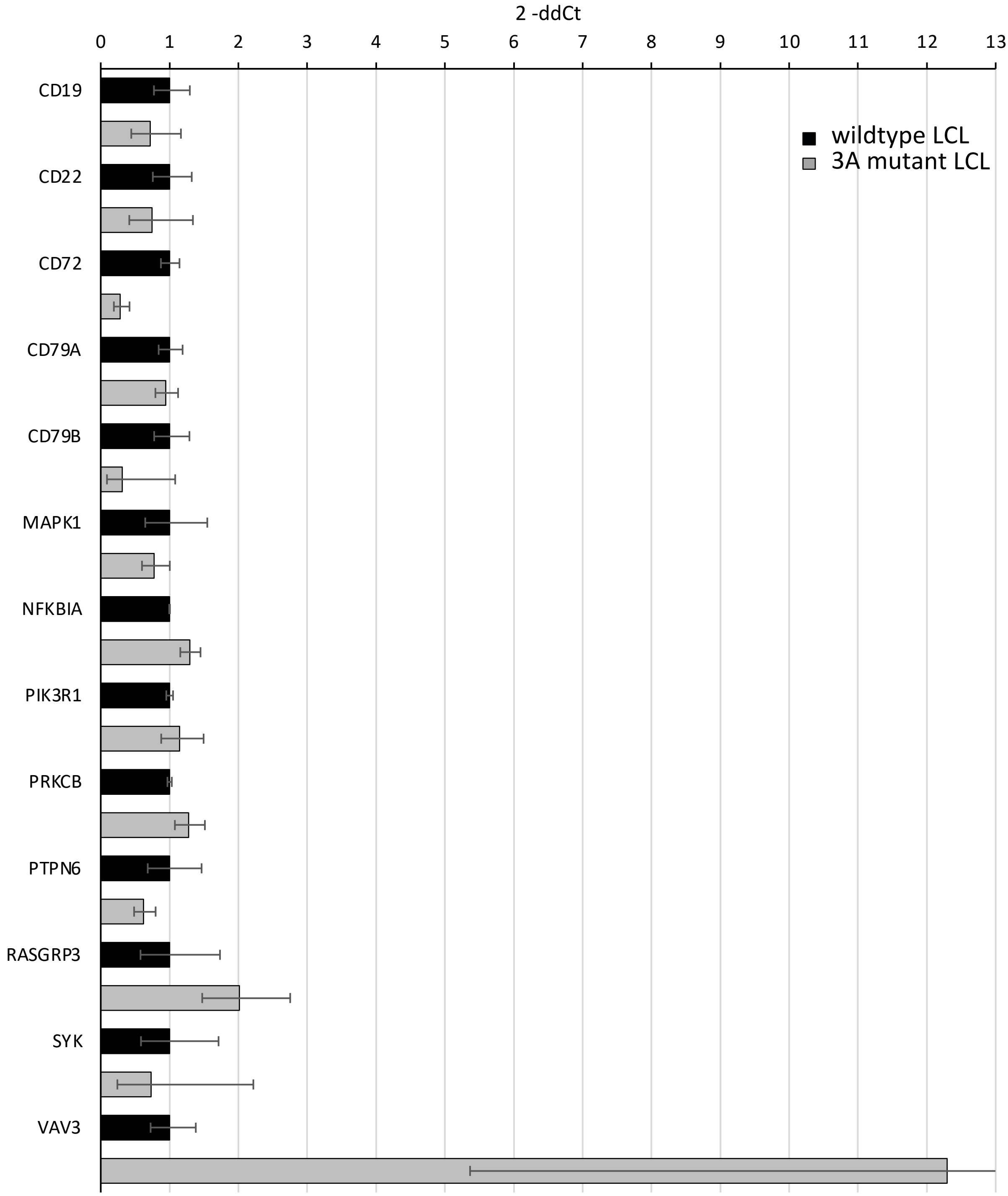

Supplementary Figure 2

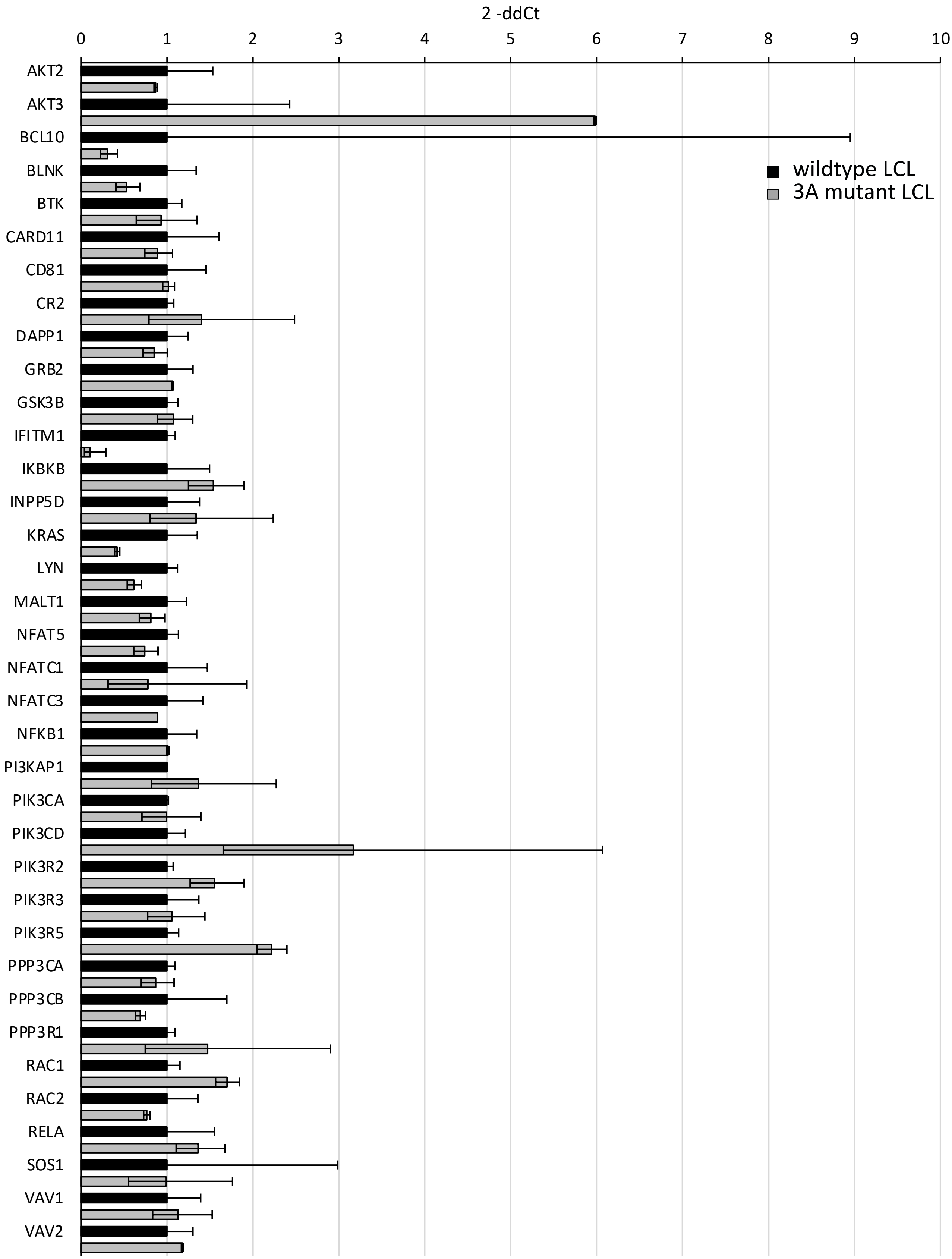

Supplementary Figure 3

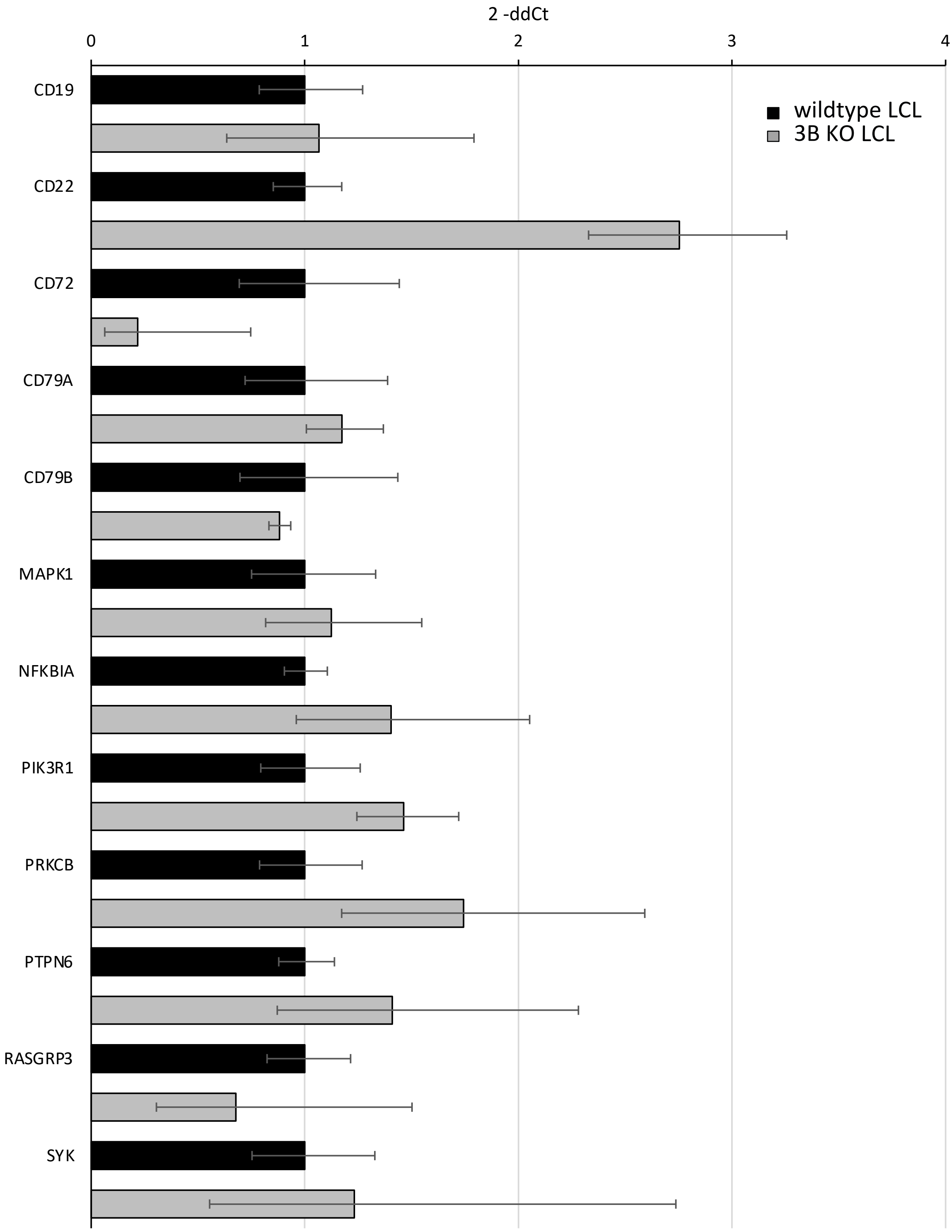

Supplementary Figure 4

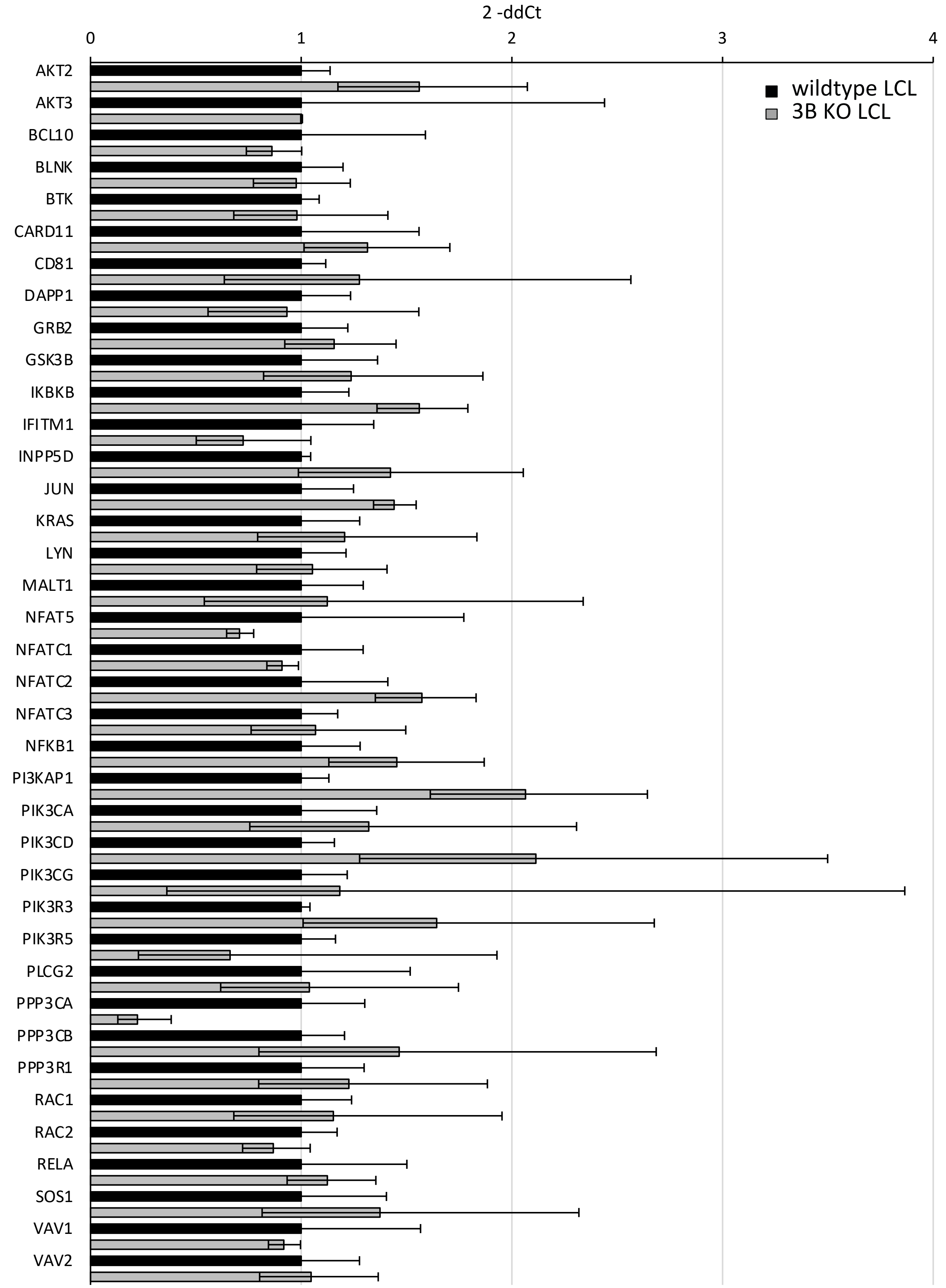

Supplementary Figure 5

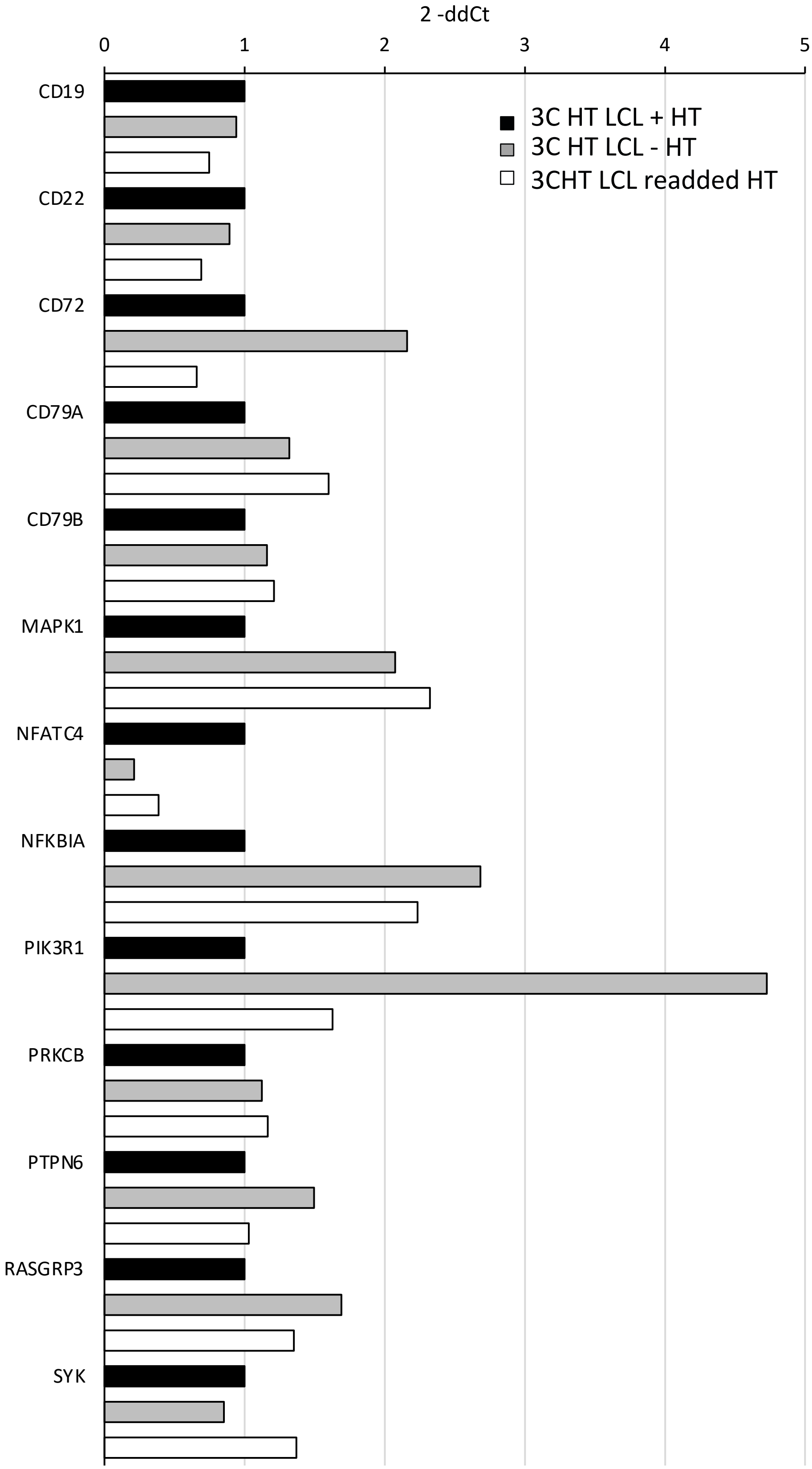

Supplementary Figure 6

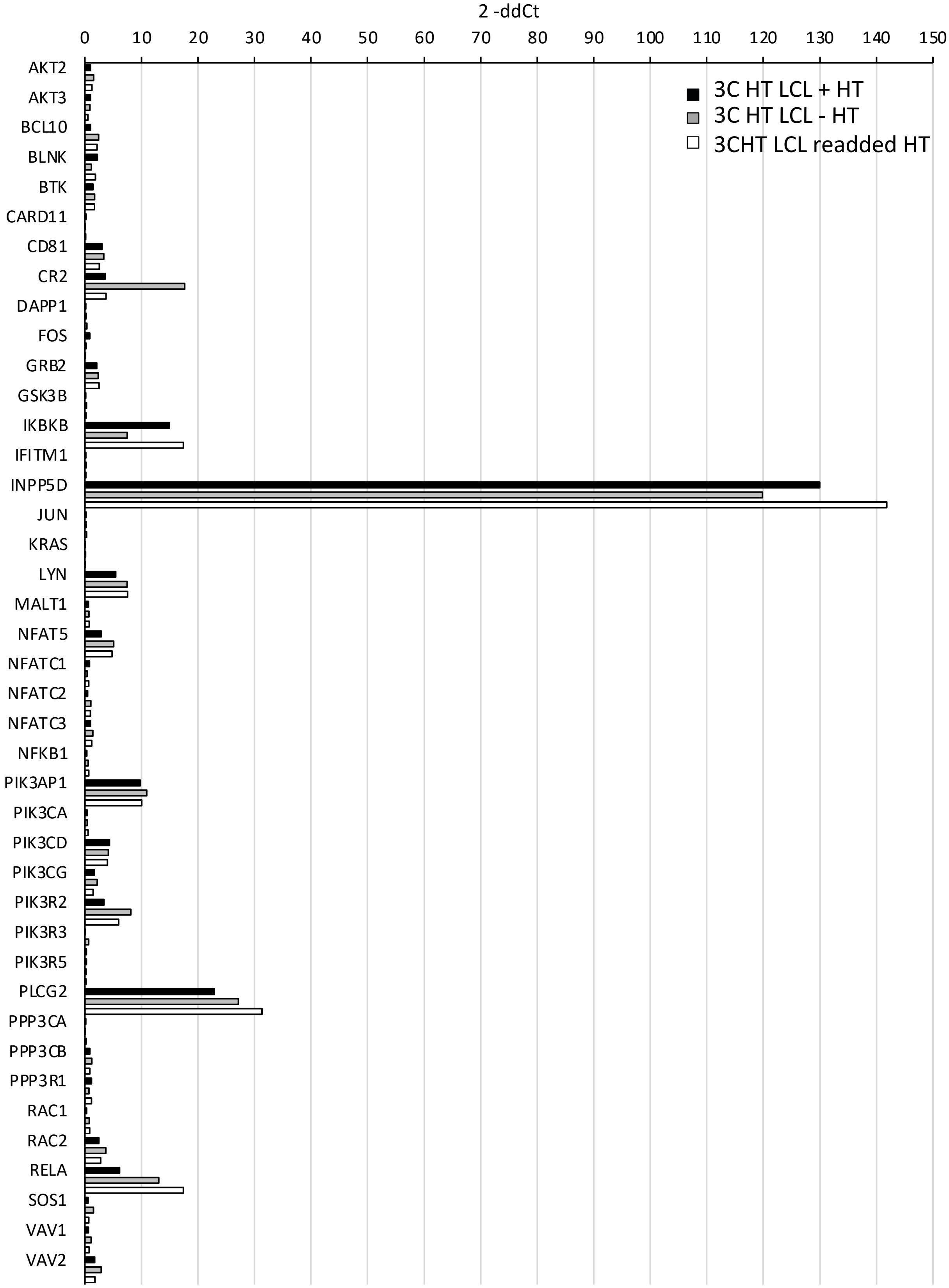

Supplementary Figure 7

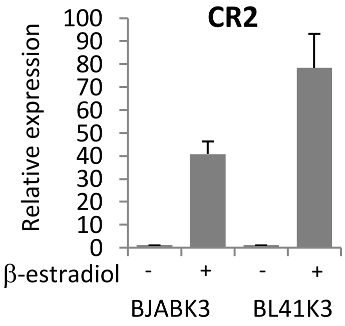

Supplementary Figure 8

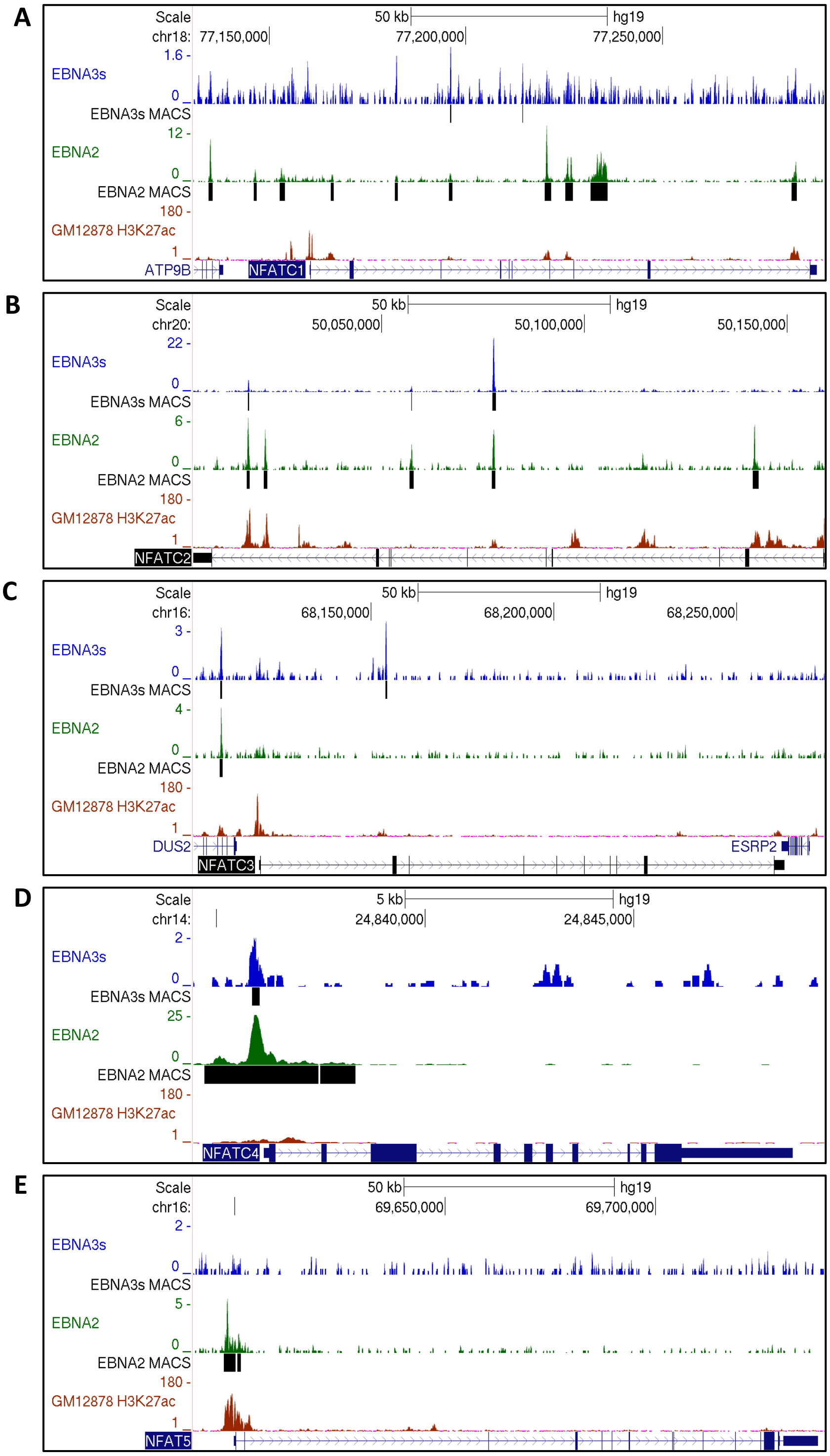

Supplementary Figure 9

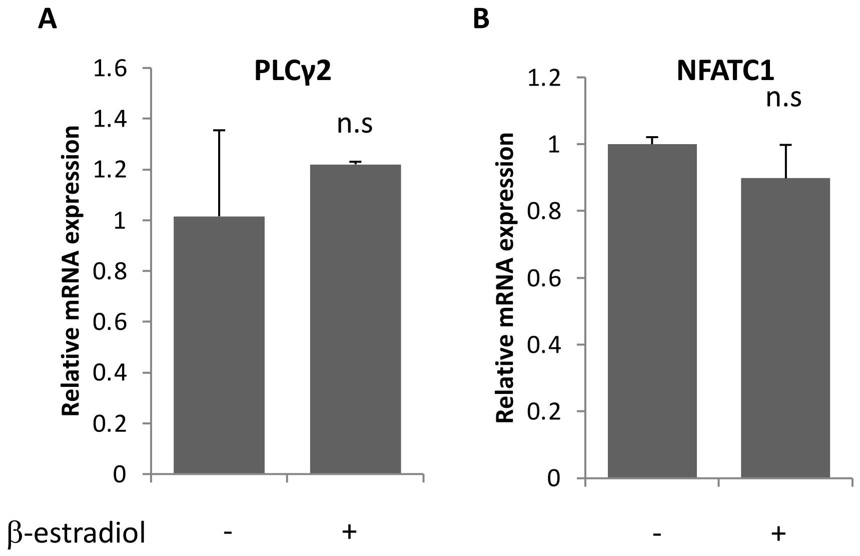

Supplementary Figure 10
